# Health-related quality of life among anaplastic lymphoma kinase (ALK)-positive non-small cell lung cancer (NSCLC) patients treated with first- and next-generation ALK tyrosine kinase inhibitors (TKIs): a systematic review and meta-analysis

**DOI:** 10.1007/s11136-025-04088-6

**Published:** 2025-11-28

**Authors:** Yan Huang, Qian Chu, Jiejun Wang, Li Zhang

**Affiliations:** 1 Department of Medical Oncology, Sun Yat-sen University Cancer Center, Guangzhou, China; 2 Department of Oncology, Tongji Hospital, Tongji Medical College, Huazhong University of Science and Technology, Wuhan, China; 3 Cancer Support and Rehabilitation Treatment Expert Committee, Chinese Society of Clinical Oncology, Beijing, China

**Keywords:** Non-Small cell lung cancer, HRQoL, ALK-TKI, Meta-analysis, Systematic review

## Abstract

**Purpose:**

To assess the possible effect of anaplastic lymphoma kinase (ALK) tyrosine kinase inhibitors (TKIs) on the health-related quality of life (HRQoL) in patients with *ALK*-positive non-small cell lung cancer (NSCLC).

**Methods:**

A systematic search was performed in PubMed, Web of Science, Embase, and ClinicalTrials.gov to identify literature published between January 2010 and January 2025. Publications reported quantitative assessments of HRQoL in *ALK*-positive NSCLC patients treated with ALK-TKIs were included. Meta-analyses were performed using random effect models.

**Results:**

A total of 805 records were identified, of which 21 were analyzed in the meta-analysis. Compared to crizotinib, next-generation ALK-TKIs showed statistically significant delayed time to deterioration (TTD) in global health status measured by the European Organization for Research and Treatment of Cancer Quality of Life Questionnaire Core 30 (EORTC QLQ-C30) (hazard ratio [HR]: 0.80; 95% confidence interval [CI]: 0.67 to 0.96). Brigatinib and alectinib demonstrated superior TTD in fatigue symptom score of EORTC QLQ-C30 compared to crizotinib (HR: 0.71; 95% CI: 0.54 to 0.92). Regarding between-arm comparisons from baseline, brigatinib and lorlatinib outperformed crizotinib in global health status, physical and emotional functioning, and symptoms scores of nausea and vomiting, fatigue, constipation, and appetite loss using EORTC QLQ-C30.

**Conclusions:**

This study is by far the most comprehensive systematic review and meta-analysis on HRQoL among *ALK*-positive NSCLC patients treated with ALK-TKIs. These findings extended prior literature by conducting a granular comparison of all available ALK-TKIs across multiple endpoints and highlighted the improved performance of next-generation ALK-TKIs in enhancing HRQoL for *ALK*-positive NSCLC patients.

**Supplementary Information:**

The online version contains supplementary material available at 10.1007/s11136-025-04088-6.

## Introduction

Lung cancer is the leading cause of cancer-related mortality worldwide and it was responsible for an estimated 1,817,469 deaths in 2022, accounting for approximately 18.7% of all cancer-related deaths globally [1]. Approximately 80–85% of all lung cancer can be classified as non-small cell lung cancer (NSCLC), of which 3–7% were identified with anaplastic lymphoma kinase (*ALK)* gene rearranged (referred as *ALK*-positive) [2, 3]. To date, eight ALK tyrosine kinase inhibitors (ALK-TKIs) are available for use: crizotinib, ceritinib, alectinib, brigatinib, ensartinib, lorlatinib, iruplinalkib (China only), and envonalkib (China only). These agents have markedly improved survival outcomes, with five-year progression-free survival rate reaching up to 60% [4], transforming the disease towards a more chronic course. As survival prolonged, the significance of patient-reported outcomes (PROs) has been increasingly acknowledged. Healthcare providers should take PROs into account when making treatment decisions to ensure the optimal provision of patient-centered care [5]. Health-related quality of life (HRQoL) is a distinct PRO measure that assesses the subjective influence of the disease and its treatment(s) on different dimensions of an individual’s daily life. These dimensions encompass physical, psychological, social functioning, and overall well-being [6].

Several validated instruments are commonly used to assess HRQoL in NSCLC research. The European Organization for Research and Treatment of Cancer Quality of Life Questionnaire Core 30 (EORTC QLQ-C30) evaluates global health status, functional domains, and symptom burden across diverse cancer populations [7]. The EORTC QLQ Lung Cancer 13 (EORTC QLQ-LC13) is a specific module designed to supplement EORTC QLQ-C30 with detailed coverage of lung cancer symptoms [8]. While the QLQ-C30 and QLQ-LC-13 provided detailed, lung cancer specific evaluation, the European Quality of Life Five Dimension (EQ-5D) offers a general measure that captures overall health status for board population [9]. In addition, the Lung Cancer Symptom Scale (LCSS) is also used to briefly measure symptom severity and impact on daily life [10].

Thus far, few meta-analyses have comprehensively evaluated HRQoL outcomes among ALK-positive NSCLC patients treated with ALK-TKIs. Previous reviews have primarily focused on survival and safety outcomes [11–14], and the few studies that assessed HRQoL included a limited number of drugs or HRQoL domains [15, 16]. To address this knowledge gap, we conducted this systematic review and meta-analysis to systematically identify, assess, and synthesize existing literature on the impact of ALK-TKIs on HRQoL in *ALK*-positive NSCLC patients.

## Materials and methods

This systematic review and meta-analysis adhered to the Preferred Reporting Items for Systematic Reviews and Meta-analyses (PRISMA) guidelines and did not require institutional review board approval [17].

### Search strategy

**Fig. 1:**
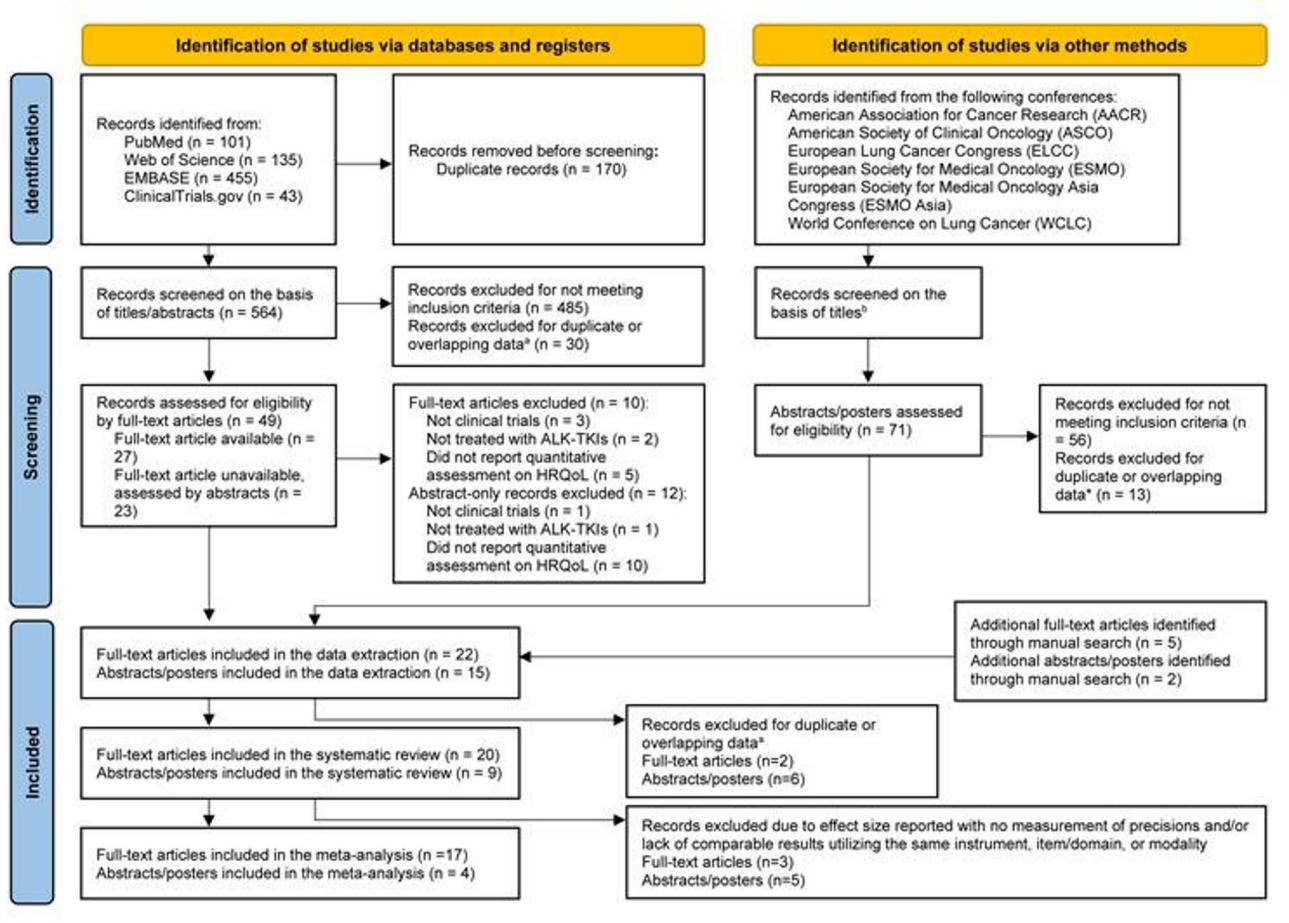
PRISMA 2020 flow diagram HRQoL: health-related quality of life; ALK-TKIs: anaplastic lymphoma kinase-tyrosine kinase inhibitors; n: number; PRISMA: Preferred Reporting Items for Systematic reviews and Meta-Analyses. ^a^Duplicate data are referred to records that were from the same clinical trial and had the same study population, study period, and follow-up length. Overlapping data are referred to records that were from the same clinical trial and had the same study population, but different study period and follow-up length. For records that had duplicate or overlapping data identified in the search, this review selected the latest full-text publications for further assessment. ^b^The number of titles screened was not counted

We searched PubMed, Web of Science, Embase, and ClinicalTrials.gov from January 1, 2010 to January 14, 2025. In addition to peer-reviewed journals, major oncology conference posters and abstracts from 2010 were screened, as shown in the PRISMA flow diagram (Fig. 1). The search strategy was developed using a combination of keywords and controlled vocabulary (e.g., Medical Subject Heading [MeSH] and Emtree) relevant to population (i.e., patients with *ALK*-positive NSCLC), intervention (i.e., ALK-TKIs) and outcomes (i.e., HRQoL). The search syntax used for each database was presented in Supplementary Table 1. Reference lists of relevant reviews were screened for potential inclusion of further studies.

### Study selection

Studies were eligible if they met the following criteria: (1) clinical trials (e.g., randomized clinical trials [RCTs], single-arm trials); (2) *ALK*-positive NSCLC patients treated with ALK-TKIs (i.e., crizotinib, ceritinib, alectinib, brigatinib, lorlatinib, ensartinib, iruplinalkib, or envonalkib); (3) reported a quantitative assessment on HRQoL. Studies were included regardless of the trial location, the comparator arm (e.g., ALK-TKIs or chemotherapy), or whether HRQoL outcomes were primary or secondary endpoints. Case studies, review articles, book chapters or dissertations were excluded. Non-English publications were also excluded due to the difficulty of ensuring accurate translation and data extraction. After removing duplicates, two reviewers independently screened and selected the articles first based on titles and abstracts and then by examining the full texts; discrepancies were resolved through discussion with a senior reviewer.

### Data extraction and quality assessment

Data extractions were conducted by two independent reviewers. Any discrepancy was resolved through discussion with a third reviewer. For each eligible study, the following information were extracted into a Microsoft® Excel sheet: publication title, the first author, publication year, study design, trial phase, trial name, study region, sample size, characteristics of patients (median age, sex, and race), type and dose of ALK-TKI treatment, ALK-TKI naiveness, treatment cycle, median follow-up time, instrument for HRQoL assessment, and quantitative findings on HRQoL impact.

We assessed the methodologic quality of each study using Cochrane Risk of Bias tool Version 2 and the assessment at each of the five domain is categorized into (1) low risk, (2) some concerns, and (3) high risk. Of note, since single-arm trials do not involve any randomization process, the first domain of the risk assessment tool were marked as “Not Applicable”. The quality assessment of each study was conducted by two independent reviewers. Any discrepancy was resolved through discussion with a third reviewer.

### Statistical analysis

The meta-analyses were performed separately for the following 4 endpoints: (1) hazard ratio (HR) for time to deterioration (TTD) in HRQoL assessment, (2) between-arm change from baseline of HRQoL scores, (3) the mean change of HRQoL scores from baseline, and (4) the differences of mean HRQoL scores at the end of observation.

A meta-analysis was performed when at least two studies reported on the same HRQoL assessment and equivalent modality of HRQoL results presentation. Data were analyzed using RStudio (version 4.1.1). Random-effects models were applied to calculate pooled estimates, given the expected heterogeneity across studies.

Statistical heterogeneity among studies was assessed using Q, *I*^2^, and τ^2^ statistics. For the *I*^*2*^ statistic, *I*^*2*^ values of 25%, 50%, and 75% were regarded as low, moderate, and high heterogeneity, respectively; and a greater value of τ^2^ indicated greater heterogeneity among the studies [18, 19]. The “meta” package in RStudio was used to conduct the meta-analysis. A 2-sided *p* < 0.05 was considered to be statistically significant.

## Results

### Search result and study selection

We identified 734 records from databases/registers, of which 49 were selected for detailed assessment. Additionally, another 71 abstracts/posters identified from conferences were assessed. Finally, a total of 20 full text articles and 9 abstracts/posters were included for systematic review and of these, 17 full text articles and 4 abstracts/posters were included for meta-analysis (Fig. 1).

### Characteristics of studies included

The characteristics of the included clinical trial studies were summarized based on 20 full-text articles and 9 abstracts/posters as shown in Table 1. Of the 20 full-text articles, 13 (65.0%) were RCT studies and 7 (35.0%) were single-arm trial studies. There were similar numbers of phase II trials (n = 9) and phase III trials (n = 11). The numbers of studies that included crizotinib, ceritinib, alectinib, brigatinib, ensartinib, and lorlatinib as the experiment arm were 5, 4, 2, 5, 1, and 3, respectively. No studies were identified that reported quantitative HRQoL results for patients treating with iruplinalkib or envonalkib. Twelve (60.0%) studies administrated ALK-TKIs in ALK-TKI naïve patients and the other 8 (40.0%) studies recruited patients who had been treated and later progressed with other ALK-TKIs. Among the 13 full-text articles on RCTs, 5 (38.5%) studies had crizotinib as the control arm, 6 (46.2%) had chemotherapy as the control arm, and another 2 (15.4%) used two different doses of brigatinib as the two interventional arms. Of the 9 abstracts/posters, seven (77.8%) studies administrated ALK-TKIs in ALK-TKI naïve patients and the other 2 (22.2%) studies recruited patients who had been treated and later progressed with other ALK-TKIs. Eight (88.88%) were phase III RCTs and one (11.11%) was phase II single-arm trial.

**Table 1:** Characteristics of the 20 full-text articles and 9 abstracts/posters

Study ID	Full-text	Trial name	Study design	Experiment arm	Control arm	Trial Phase	ALK-TKI naïve	Sample size	Instrument for HRQoL assessment	Modality of HRQoL results presentation
Shaw et al. [33]	Yes	PROFILE 1007	RCT	Crizotinib (250 mg)	Chemotherapy (pemetrexed or docetaxel)	III	Yes	Crizotinib 250 mg: 161^a^ Chemotherapy: 150^a^	EORTC-QLQ-C30 EORTC-QLQ-LC13	Time to deterioration (symptoms), change from baseline (global), change from baseline (functioning), change from baseline (symptoms)
Blackhall et al. [45]	Yes	PROFILE 1007	RCT	Crizotinib (250 mg)	Chemotherapy (pemetrexed or docetaxel)	III	Yes	Crizotinib 250 mg: 162^a^ Chemotherapy: 151^a^	EORTC-QLQ-C30 EORTC-QLQ-LC13 EQ-5D	Between-arm change from baseline (global), between-arm change from baseline (functioning), between-arm change from baseline (symptoms), descriptive proportion of improvement/stability/deterioration (global), descriptive proportion of improvement/stability/deterioration (functioning), descriptive proportion of improvement/stability/deterioration (symptoms), time to deterioration (symptoms), mean HRQoL score (global), change from baseline (global)
Solomon et al. [25]	Yes	PROFILE 1014	RCT	Crizotinib (250 mg)	Chemotherapy (pemetrexed − cisplatin or pemetrexed − carboplatin)	III	Yes	Crizotinib 250 mg: 172 Chemotherapy: 171	EORTC-QLQ-C30 EORTC-QLQ-LC13 EQ-5D	Change from baseline (global), change from baseline (functioning), change from baseline (symptoms), time to deterioration (symptoms)
Blackhall et al. [46]	Yes	PROFILE 1005	Single-arm	Crizotinib (250 mg)	–	II	Yes	Crizotinib 250 mg: 976^a^	EORTC-QLQ-C30 EORTC-QLQ-LC13 EQ-5D	Descriptive proportion of improvement/stability/deterioration (global), descriptive proportion of improvement/stability/deterioration (functioning), descriptive proportion of improvement/stability/deterioration (symptoms)
Wu et al. [47]	Yes	PROFILE 1029	RCT	Crizotinib (250 mg)	Chemotherapy (pemetrexed plus cisplatin, or carboplatin)	III	Yes	Crizotinib 250 mg: 104^b^ Chemotherapy: 103^b^	EORTC-QLQ-C30 EORTC-QLQ-LC13 EQ-5D	Between-arm change from baseline (global), between-arm change from baseline (functioning), between-arm change from baseline (symptoms), time to deterioration (symptoms), mean treatment difference (global), mean treatment difference (symptoms)
Soria et al*.* 2017 [48]	Yes	ASCEND-4	RCT	Ceritinib (750 mg)	Chemotherapy (cisplatin, or carboplatin plus pemetrexed)	III	Yes	Ceritinib 750 mg: 189 Chemotherapy: 187	EORTC-QLQ-C30 EORTC-QLQ-LC13 LCSS EQ-5D	Between-arm change from baseline (global), between-arm change from baseline (functioning), between-arm change from baseline (symptoms), time to deterioration (symptoms)
Nishio et al. [49]	Yes	ASCEND-3	Single-arm	Ceritinib (750 mg)	–	II	Yes	Ceritinib 750 mg: 124	EORTC-QLQ-C30 EORTC-QLQ-LC13 LCSS	Change from baseline (global), change from baseline (symptoms)
Perol et al. [50]	Yes	ALEX	RCT	Alectinib (600 mg)	Crizotinib (250 mg)	III	Yes	Alectinib 600 mg: 100^a^ Crizotinib 250 mg: 97^a^	EORTC-QLQ-C30 EORTC-QLQ-LC13	Time to deterioration (global), time to deterioration (functioning), time to deterioration (symptoms), change from baseline (symptoms), descriptive proportion of improvement/stability/deterioration (global), descriptive proportion of improvement/stability/deterioration (functioning), descriptive proportion of improvement/stability/deterioration (symptoms)
Camidge et al. [21]	Yes	ALTA-1L	RCT	Brigatinib (90- > 180 mg)	Crizotinib (250 mg)	III	Yes	Brigatinib 90- > 180 mg: 137 Crizotinib 250 mg: 138	EORTC-QLQ-C30 EORTC-QLQ-LC13	Time to deterioration (global), time to deterioration (functioning), time to deterioration (symptoms)
Garcia Campelo et al. [51]	Yes	ALTA-1L	RCT	Brigatinib (90- > 180 mg)	Crizotinib (250 mg)	III	Yes	Brigatinib 90- > 180 mg: 131^a^ Crizotinib 250 mg: 131^a^	EORTC-QLQ-C30 EORTC-QLQ-LC13	Time to deterioration (global), time to deterioration (functioning), time to deterioration (symptoms), change from baseline (global), change from baseline (functioning), change from baseline (symptoms), duration of improvement (global), descriptive proportion of improvement/stability/deterioration (global), descriptive proportion of improvement/stability/deterioration (symptoms)
Mazieres et al. [52]	Yes	CROWN	RCT	Lorlatinib (100 mg)	Crizotinib (250 mg)	III	Yes	Lorlatinib 100 mg: 148^a^ Crizotinib 250 mg: 140^a^	EORTC-QLQ-C30 EORTC-QLQ-LC13	Time to deterioration (symptoms), between-arm change from baseline (global), between-arm change from baseline (functioning), between-arm change from baseline (symptoms)
Solomon et al. [52]	Yes	CROWN	RCT	Lorlatinib (100 mg)	Crizotinib (250 mg)	III	Yes	Lorlatinib 100 mg: 148^a^ Crizotinib 250 mg: 139^a^	EORTC-QLQ-C30 EORTC-QLQ-LC13	Time to deterioration (symptoms), between-arm change from baseline (global), between-arm change from baseline (functioning), between-arm change from baseline (symptoms)
Crino et al. [53]	Yes	ASCEND-2	Single-arm	Ceritinib (750 mg)	–	II	No	Ceritinib 750 mg: 140	EORTC-QLQ-C30 EORTC-QLQ-LC13 LCSS	Change from baseline (global), change from baseline (functioning), change from baseline (symptoms)
Shaw et al. [20]	Yes	ASCEND-5	RCT	Ceritinib (750 mg)	Chemotherapy (pemetrexed or docetaxel)	III	No	Ceritinib 750 mg: 115 Chemotherapy: 116	EORTC-QLQ-C30 EORTC-QLQ-LC13 LCSS EQ-5D	Time to deterioration (symptoms), mean treatment difference (global)
Ou et al. [54]	Yes	NP28761	Single-arm	Alectinib (600 mg)	–	II	No	Alectinib 600 mg: 87	EORTC-QLQ-C30 EORTC-QLQ-LC13	Change from baseline (global), change from baseline (functioning), change from baseline (symptoms), time to deterioration (symptoms), time to improvement (symptoms)
Kawata et al. [55]	Yes	ALTA	RCT	Brigatinib (90 mg)	Brigatinib (90- > 180 mg)	II	No	Brigatinib 90 mg: 105^a^ Brigatinib 90- > 180 mg: 103^a^	EORTC-QLQ-C30 EQ-5D	Change from baseline (global), mean HRQoL score (global)
Lenderking et al. [56]	Yes	ALTA	RCT	Brigatinib (90 mg)	Brigatinib (90- > 180 mg)	II	No	Brigatinib 90 mg: 105^a^ Brigatinib 90- > 180 mg: 103^a^	EORTC-QLQ-C30	Change from baseline (global), mean HRQoL score (global), mean HRQoL score (symptoms), descriptive proportion of improvement/stability/deterioration (global)
Ou et al. [57]	Yes	ALTA-2	Single-arm	Brigatinib (90- > 180 mg)	–	II	No	Brigatinib 90- > 180 mg: 103	EORTC-QLQ-C30 EORTC-QLQ-LC13	Change from baseline (global), change from baseline (symptoms)
Yang et al. [58]	Yes	NCT03215693	Single-arm	Ensartinib (225 mg)	–	II	No	Ensartinib 225 mg: 156	EORTC-QLQ-C30 EORTC-QLQ-LC13 LCSS	Change from baseline (global), change from baseline (functioning), change from baseline (symptoms)
Solomon et al. [59]	Yes	NCT01970865	Single-arm	Lorlatinib (100 mg)	–	II	No	Lorlatinib 100 mg: 184^a^	EORTC-QLQ-C30 EORTC-QLQ-LC13	Descriptive proportion of improvement/stability/deterioration (global), descriptive proportion of improvement/stability/deterioration (functioning), descriptive proportion of improvement/stability/deterioration (symptoms)
Felip et al. [60]	No	PROFILE 1014	RCT	Crizotinib (250 mg)	Chemotherapy (pemetrexed plus cisplatin or carboplatin)	III	Yes	Crizotinib 250 mg: 172 Chemotherapy: 171	EQ-5D	Mean HRQoL score (global)
Blackhall et al. [61]	No	PROFILE 1014	RCT	Crizotinib (250 mg)	Chemotherapy (pemetrexed plus cisplatin or carboplatin)	III	Yes	Crizotinib 250 mg: 172 Chemotherapy: 171	EORTC-QLQ-C30 EORTC-QLQ-LC13	Descriptive proportion of improvement/stability/deterioration (global), descriptive proportion of improvement/stability/deterioration (functioning), descriptive proportion of improvement/stability/deterioration (symptoms)
Blackhall et al. [62]	No	PROFILE 1007	RCT	Crizotinib (250 mg)	Chemotherapy (pemetrexed or docetaxel)	III	Yes	Crizotinib 250 mg: 172 Chemotherapy: 171	EQ-5D	Mean HRQoL score (global), change from baseline (global)
Blackhall et al. [63]	No	PROFILE 1007	RCT	Crizotinib (250 mg)	Chemotherapy (pemetrexed or docetaxel)	III	Yes	Crizotinib 250 mg: 172 Chemotherapy: 171	EORTC-QLQ-C30 EORTC-QLQ-LC13	Descriptive proportion of improvement/stability/deterioration (global), descriptive proportion of improvement/stability/deterioration (functioning), descriptive proportion of improvement/stability/deterioration (symptoms), relative risk ratios for improvement rates (symptoms)
Zhou et al. [64]	No	ALESIA	RCT	Alectinib (600 mg)	Crizotinib (250 mg)	III	Yes	Alectinib 600 mg: 125^b^ Crizotinib 250 mg: 62^b^	EORTC-QLQ-C30 EORTC-QLQ-LC13	Time to deterioration (global), time to deterioration (symptoms),
Selvaggi et al. [65]	No	eXalt3	RCT	Ensartinib (225 mg)	Crizotinib (250 mg)	III	Yes	Ensartinib 225 mg: 143 Crizotinib 250 mg: 147	EORTC-QLQ-C30	Time to deterioration (global)
Liu et al. [66]	No	CROWN	RCT	Lorlatinib (100 mg)	Crizotinib (250 mg)	III	Yes	Lorlatinib 100 mg: 148^a^ Crizotinib 250 mg: 140^a^	EQ-5D	Mean HRQoL score (global), mean treatment difference (global), change from baseline (global)
Mazieres, et al. [67]	No	ALUR	RCT	Alectinib (600 mg)	Chemotherapy (pemetrexed or docetaxel)	III	No	Alectinib 600 mg: 72 Chemotherapy: 35	EORTC-QLQ-C30 EORTC-QLQ-LC13	Time to deterioration (symptoms), change from baseline (functioning), change from baseline (symptoms), descriptive proportion of improvement/stability/deterioration (functioning)
Greillier et al. [68]	No	ATALK	Single-arm	Alectinib (600 mg)	–	II	No	Alectinib 600 mg: 43^a^	EORTC-QLQ-C30 EORTC-QLQ-LC13 EQ-5D	Mean HRQoL score (global), time to deterioration (global), time to deterioration (functioning), time to deterioration (symptoms), descriptive proportion of improvement/stability/deterioration (global)

ALK-TKI, Anaplastic lymphoma kinase-tyrosine kinase inhibitor; EORTC QLQ-LC13, European Organization for Research and Treatment of Cancer Quality of Life Questionnaire Lung Cancer 13; EORTC QLQ-C30, European Organization for Research and Treatment of Cancer Quality of Life Questionnaire Core 30; EQ-5D, European Quality of Life Five Dimension; HRQoL, health-related quality of life; LCSS: Lung Cancer Symptom Scale; RCT, Randomized clinical trial

^a^PRO-evaluable sample size

^b^Reported study population is Asian

The main characteristics of 20 full-text articles and 9 abstracts/posters included were presented in Table 1. The main characteristics extracted were trial name, study design, experiment arm, control arm, trial phase, whether ALK-TKI naïve, sample size, instrument for HRQoL assessment, and modality of HRQoL results presentation. Most studies assessed HRQoL using EORTC QLQ-C30, often in combination with the lung cancer-specific QLQ-LC13 module. A full list of HRQoL measures across studies was provided in Table 1. In addition, HRQoL instrument measured and reported, modality of HRQoL results presentation, and number of modalities of HRQoL results presentation were shown in Supplementary Table 2.

### Quality assessment

The results of quality assessment of all 20 full-text articles included in the systematic review were shown in Supplementary Table 3. Overall, the quality of the included studies was low, mainly due to high risk of bias in selection of the reported result and some concerns of bias in measurement of outcome, despite low risk of bias in other domains. Of the 20 full-text articles, 7 were identified as with some concerns of bias and 13 had high risk of bias.

Eight records were excluded from meta-analysis because the effect sizes were reported with no measurement of precisions, measured instrument and symptoms not specified and/or lack of comparable results utilizing the same instrument, item and domain, or modality.

### Results of meta-analyses

#### Meta analysis of HRQoL comparing next-generation ALK-TKIs to crizotinib

##### Meta-analysis of global health status of TTD comparing next-generation ALK-TKIs to crizotinib

**Fig. 2:**
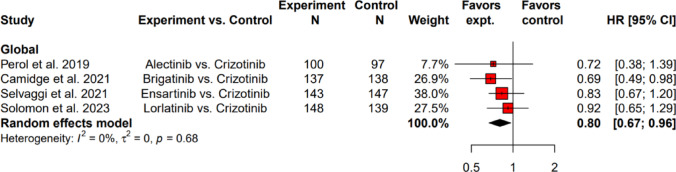
Hazard ratios for time to deterioration of global health status score comparing next-generation ALK-TKIs vs. crizotinib as first-line ALK-TKI using EORTC QLQ-C30. ALK-TKI: anaplastic lymphoma kinase-tyrosine kinase inhibitor; Cl: confidence level; EORTC QLQ-C30: European Organization for Research and Treatment of Cancer Quality of Life Questionnaire Core 30; expt: experiment; HR: hazard ratio; N: number

Among ALK-TKI naïve patients, the results of the meta-analysis for the HR of TTD in HRQoL assessment revealed that next-generation ALK-TKIs were associated with a statistically significant delayed TTD in global health status of EORTC QLQ-C30 (HR: 0.80; 95% CI: 0.67 to 0.96), compared to crizotinib (Fig. 2). There was no between-study heterogeneity (*I*^2^ = 0%, τ^2^ = 0, *p* = 0.68). Of these four next-generation ALK-TKIs, brigatinib was the only one showed a statistically significant delayed TTD in in global health status of EORTC QLQ-C30 (HR: 0.69; 95% CI: 0.49 to 0.98), compared to crizotinib. Ceritinib was not included due to the lack of study reporting TTD in global health status using EORTC QLQ-C30 compared with crizotinib.

Additionally, median TTD in months for global health status using EORTC QLQ-C30 was reported for brigatinib (26.7; 95% CI: 8.3 to not estimable) vs. crizotinib (8.3; 95% CI: 5.7 to 13.5), lorlatinib (24.0; 95% CI: 6.5 to not estimable) vs. crizotinib (12.0; 95% CI: 6.5 to not estimable) and alectinib (15.0; 95% CI: 6.3 to not estimable). Meta-analysis was not performed since the above three studies’ upper bound of 95% CIs were not estimable.

##### Meta-analysis of functioning scores of TTD comparing next-generation ALK-TKIs to crizotinib

**Fig. 3:**
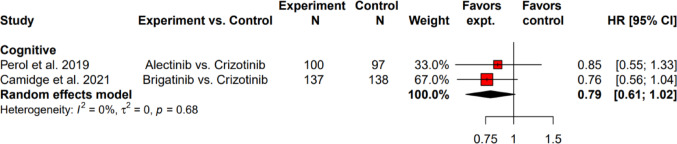
Hazard ratios for time to deterioration of functioning score comparing alectinib and brigatinib to crizotinib as first-line ALK-TKIs using EORTC QLQ-C30. ALK-TKI: anaplastic lymphoma kinase-tyrosine kinase inhibitor; Cl: confidence level; EORTC QLQ-C30: European Organization for Research and Treatment of Cancer Quality of Life Questionnaire Core 30; expt: experiment; HR: hazard ratio; N: number

The EORTC QLQ-C30 comprises five domains related to functioning: social, role, physical, cognitive, and emotional. Meta-analysis was performed for cognitive functioning as only one study reported the analyzable results of the other four functioning domains. The pooled HR for TTD in cognitive functioning for alectinib and brigatinib was 0.79 (95% CI: 0.61 to 1.02) and there was no between-study heterogeneity (*I*^2^ = 0%, τ^2^ = 0, *p* = 0.68) (Fig. 3).

##### Meta-analysis of symptom scores of TTD comparing next-generation ALK-TKIs to crizotinib

**Fig. 4:**
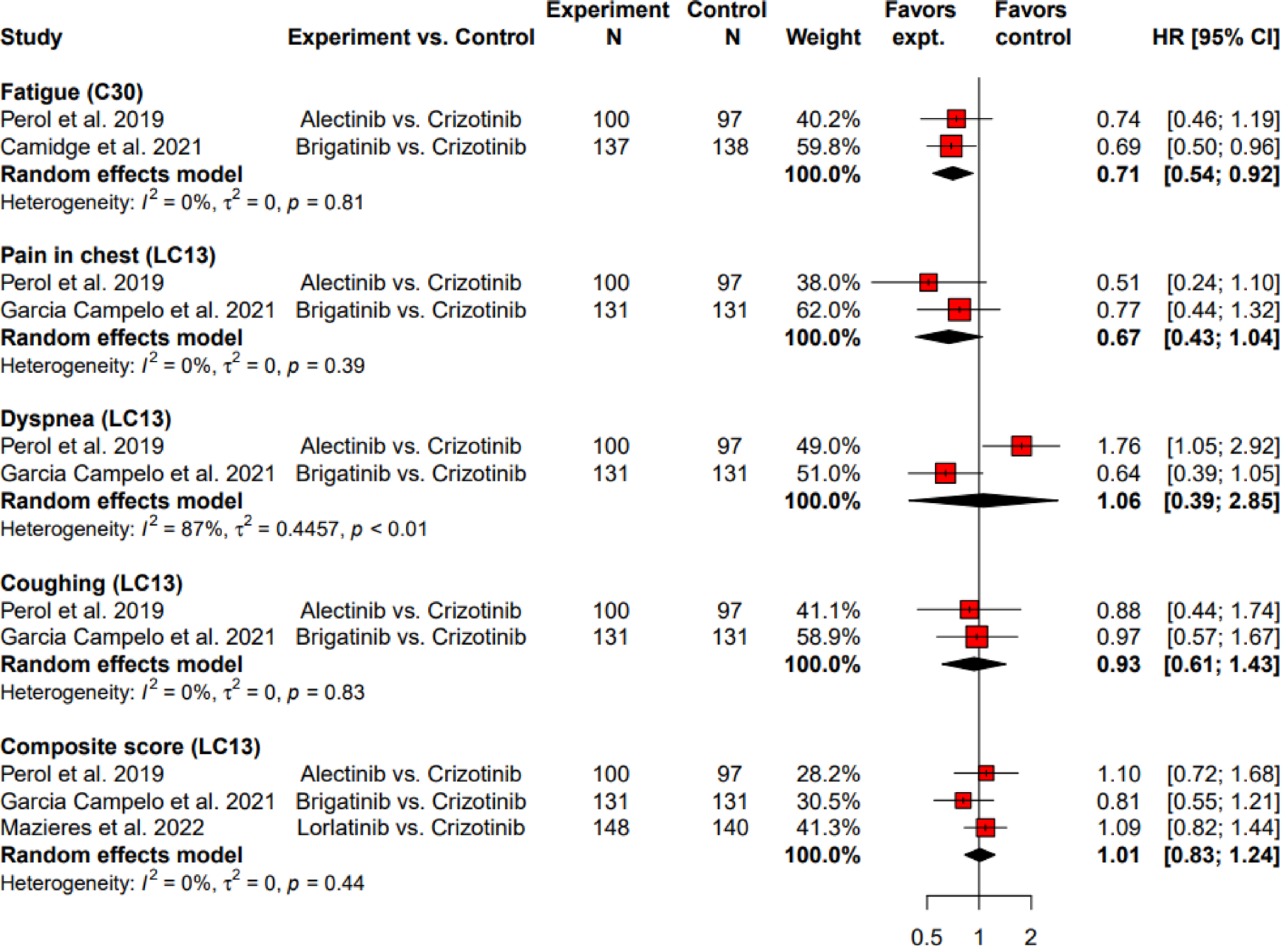
Hazard ratios for time to deterioration of symptom score comparing next generation ALK-TKIs to crizotinib as first-line ALK-TKI using EORTC QLQ-C30 and EORTC QLQ-LC13. ALK-TKI: anaplastic lymphoma kinase-tyrosine kinase inhibitor; Cl: confidence level; EORTC QLQ-C30: European Organization for Research and Treatment of Cancer Quality of Life Questionnaire Core 30; EORTC QLQ-LC13: EORTC QLQ-Lung Cancer 13; expt: experiment; HR: hazard ratio; N: number

The pooled TTD was significantly longer with alectinib and brigatinib than with crizotinib in fatigue symptom assessed with EORTC QLQ-C30, with a HR of 0.71 (95% CI: 0.54 to 0.92), and the heterogeneity was not significant (*I*^2^ = 0%, τ^2^ = 0, *p* = 0.81) (Fig. 4). As for symptoms assessed with EORTC QLQ-LC13, none of the pooled analyses on HRs of TTD in pain in chest, dyspnea, coughing, and composite score showed significant direction of favoring (Fig. 4).

##### Meta-analysis of global health status of between-arm change from baseline comparing next-generation ALK-TKIs to crizotinib

**Fig. 5:**
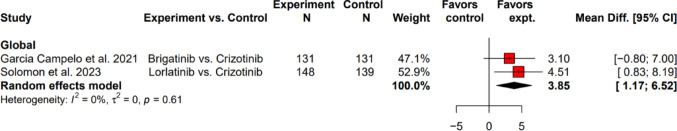
Between-arm change from baseline of global health status score comparing brigatinib and lorlatinib to crizotinib as first-line ALK-TKIs using EORTC QLQ-C30. ALK-TKI: anaplastic lymphoma kinase-tyrosine kinase inhibitor; Cl: confidence level; EORTC QLQ-C30: European Organization for Research and Treatment of Cancer Quality of Life Questionnaire Core 30; expt: experiment; HR: hazard ratio; N: number

Two studies presented the between-arm change from baseline of global health status in HRQoL using EORTC QLQ-C30 as shown in Fig. 5. Compared to crizotinib, the pooled mean differences were 3.85 (95% CI: 1.17 to 6.52), favoring brigatinib and lorlatinib over crizotinib. The heterogeneity was not significant for estimates (*I*^2^ = 0%, τ^2^ = 0, *p* = 0.61). Lorlatinib demonstrated significantly greater between-arm change from baseline in global health status compared to crizotinib (MD = 4.51; 95% CI: 0.83 to 8.19).

##### Meta-analysis of functioning scores of between-arm change from baseline comparing next-generation ALK-TKIs to crizotinib

**Fig. 6:**
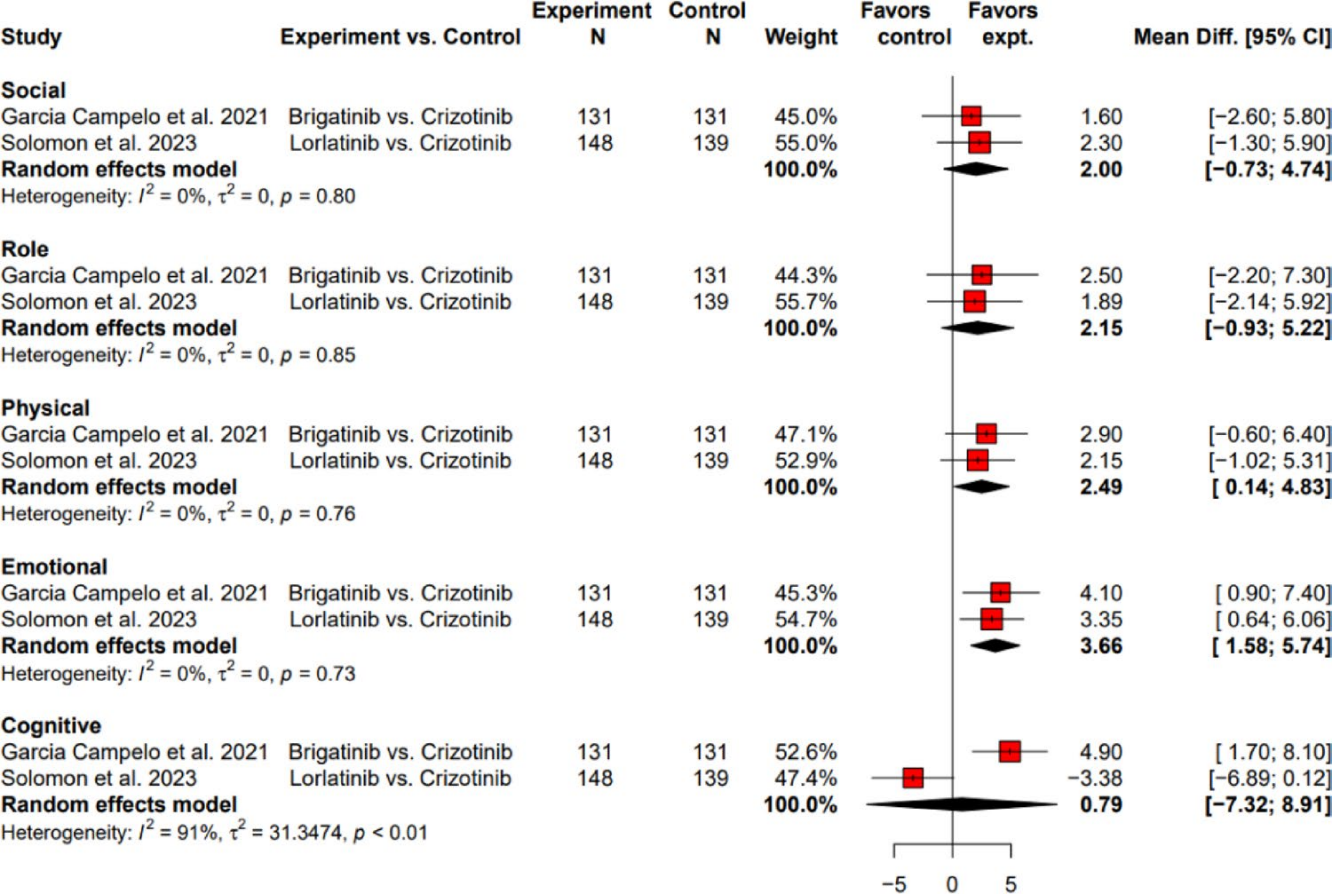
Between-arm change from baseline of functioning score comparing brigatinib and lorlatinib to crizotinib as first-line ALK-TKIs using EORTC QLQ-C30. ALK-TKI: anaplastic lymphoma kinase-tyrosine kinase inhibitor; Cl: confidence level; EORTC QLQ-C30: European Organization for Research and Treatment of Cancer Quality of Life Questionnaire Core 30; expt: experiment; HR: hazard ratio; N: number

Two studies reported comparable HRQoL in functioning using EORTC QLQ-C30 with crizotinib as control, as shown in Fig. 6. The pooled between-arm change from baseline for physical and emotional functioning were 2.49 (95% CI: 0.14 to 4.83) and 3.66 (95% CI: 1.58 to 5.74), respectively, favoring brigatinib and lorlatinib over crizotinib. There was no statistical significance among the pooled results of social, role, and cognitive functioning when comparing brigatinib and lorlatinib with crizotinib. However, for cognitive functioning, brigatinib demonstrated significant improvement compared to crizotinib (MD, 4.90; 95% CI: 1.70 to 8.10), while lorlatinib presented worsening (MD, − 3.38; 95% CI: − 6.89 to 0.12), though the result was not statistically significant. The heterogeneity of functioning scores were not significant for estimates except for cognitive functioning (*I*^2^ = 91%, τ^2^ = 31.35, *p* < 0.01).

##### Meta-analysis of symptom scores of between-arm change from baseline comparing next-generation ALK-TKIs to crizotinib

**Fig. 7:**
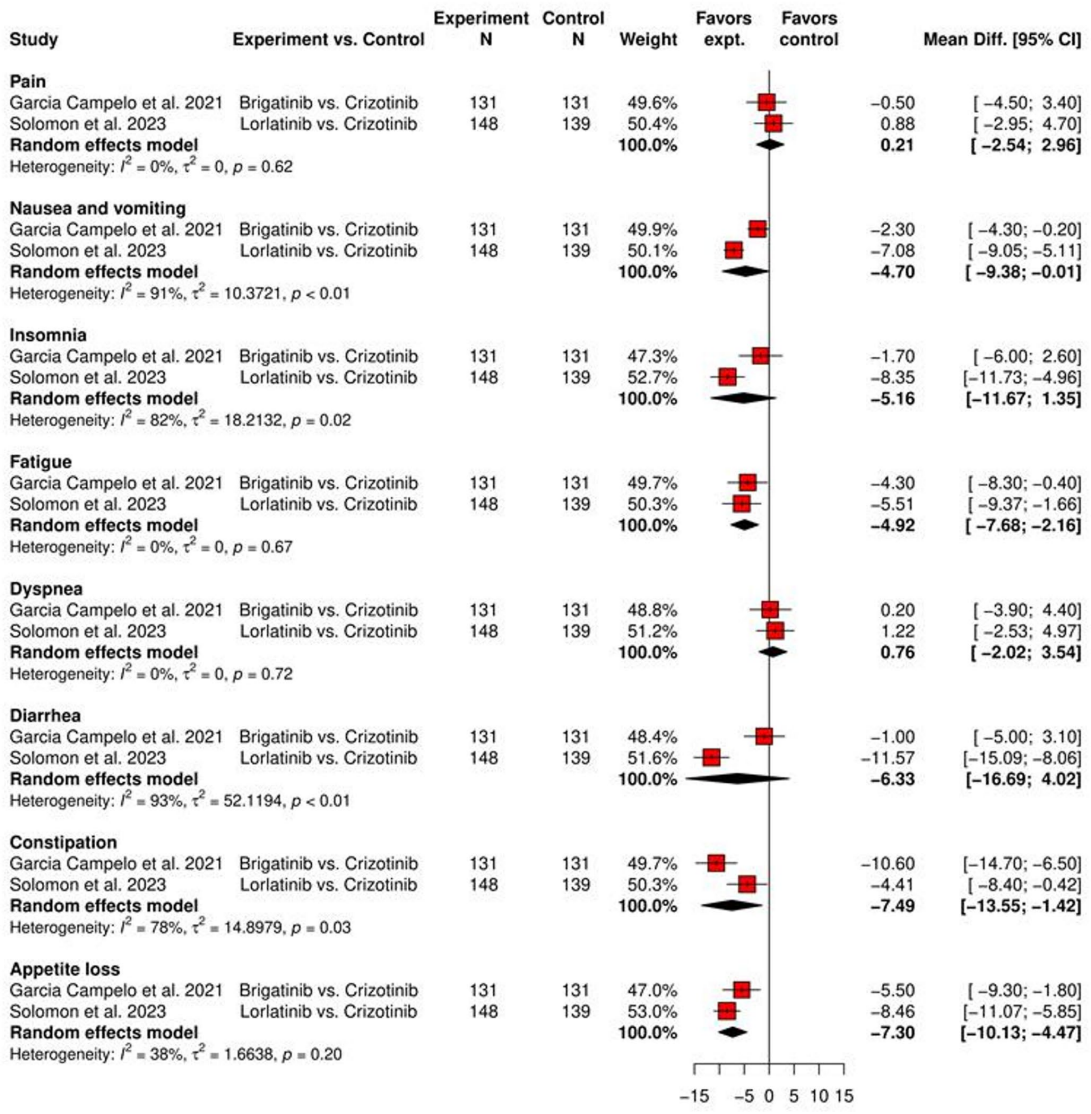
Between-arm change from baseline of symptom score comparing brigatinib and lorlatinib to crizotinib as first-line ALK-TKIs using EORTC QLQ-C30. ALK-TKI: anaplastic lymphoma kinase-tyrosine kinase inhibitor; Cl: confidence level; EORTC QLQ-C30: European Organization for Research and Treatment of Cancer Quality of Life Questionnaire Core 30; expt: experiment; HR: hazard ratio; N: number

Two studies reported comparable between-arm change from baseline of HRQoL in eight symptoms using EORTC QLQ-C30 with crizotinib as control, as shown in Fig. 7. The pooled between-arm change from baseline of nausea and vomiting, fatigue, constipation and appetite loss symptoms were − 4.70 (95% CI: − 9.38 to − 0.01), − 4.92 (95% CI: − 7.68 to − 2.16), − 7.49 (95% CI: − 13.55 to − 1.42), and − 7.30 (95% CI: − 10.13 to − 4.47), respectively, indicating a significant improvement in favor of brigatinib and lorlatinib over crizotinib. The pooled results of pain, insomnia, dyspnea and diarrhea symptoms were not significant. Significant heterogeneities were found among estimates of nausea and vomiting (*I*^2^ = 91%, τ^2^ = 10.37, *p* < 0.01), insomnia (*I*^2^ = 82%, τ^2^ = 18.21, *p* = 0.02), diarrhea (*I*^2^ = 94%, τ^2^ = 57.14, *p* < 0.01), and constipation (*I*^2^ = 78%, τ^2^ = 14.90, *p* = 0.03).

#### Meta-analysis of HRQoL of ALK-TKIs

In terms of endpoint of change from baseline, the pooled global health status scores in EORTC QLQ-C30 for crizotinib, ceritinib, alectinib and brigatinib significantly improved after treatment (Supplementary Fig. 1). As for symptom scores, the pooled results of change from baseline in pain in chest and coughing (EORTC QLQ-LC13) were significantly alleviated after treating with alectinib, brigatinib or ceritinib (Supplementary Fig. 2). Supplementary Fig. 3 presented the pooled results of change from baseline assessed by EQ-5D utility index scores showed significant health status improvement with crizotinib, brigatinib and lorlatinib.

#### Meta analysis of HRQoL comparing ALK-TKIs to chemotherapy

The comparison between crizotinib and ceritinib to chemotherapy demonstrated significant improved pooled effects of global health status, functioning (i.e., social, role, physical, emotional, and cognitive) and symptom scores (i.e., pain, insomnia, fatigue, dyspnea, and appetite loss) as measured by the between-arm change from baseline using EORTC QLQ-C30 (Supplementary Figs. 4, 5, 6). Additionally, the symptom scores measured by the between-arm change from baseline using EORTC QLQ-LC13 and TTD using EORTC QLQ-LC13 and LCSS were detailed in Supplementary Figs. 7 and 8 respectively. Specifically, the pooled analyses indicated that crizotinib and ceritinib significantly enhanced symptom scores of sore mouth, pain in chest, pain in arm or shoulder, dyspnea, dysphagia, coughing, and composite score, compared with chemotherapy. In addition, the pooled analyses of differences in mean visual analog scale scores and utility index scores using EQ-5D showed that crizotinib and ceritinib significantly improved the overall HRQoL (Supplementary Fig. 9).

## Discussion

Given that the median survival time of patients treated with ALK-TKIs has been greatly prolonged [20–25], the treatment goal has transformed from only extending their lifespan to promoting their overall well-being. While evaluating efficacy and safety in clinical trials is crucial, it is equally important to consider the impact of treatments on patients’ quality of life. To our best knowledge, this is the most comprehensive systematic review and meta-analysis to evaluate the HRQoL among *ALK*-positive NSCLC patients treated with ALK-TKIs. We systematically assessed 29 publications from 16 distinct trials that employed different HRQoL instruments and reported various categories of modalities. Out of these, 21 publications were included in the meta-analysis. Overall, these meta-analyzed results provided evidence that next-generation ALK-TKIs demonstrated improved HRQoL compared to crizotinib. Additionally, this study identified an overall lack of assessment on HRQoL in ALK-TKIs trials.

Specifically, as the first-line ALK therapies, next-generation ALK-TKIs, including alectinib, brigatinib, ensartinib and lorlatinib, demonstrated an improved global health status compared with crizotinib. The pooled between-arm change from baseline also showed improvements in brigatinib and lorlatinib over crizotinib. The results were consistent with a real-world study by Tse et al*.* which indicated that alectinib, brigatinib, and lorlatinib exhibited high mean EQ-5D-derived health utility scores longitudinally in the real-world setting [16]. A previous prospective non-interventional study reported that the association with deterioration of global health status of HRQoL and disease progression was most pronounced in lung cancer with the 6.5 points of score (95% CI: 3.5 to 9.9; *p* < 0.001) [26]. Therefore, the observed improvement in global HRQoL for next-generation ALK-TKIs in our study may be associated with their prolonged progression-free survival. This aligns with pivotal trials including ALEX, ALTA-1L, eXalt3 and CROWN [4, 21, 22, 24], where alectinib, brigatinib, ensartinib and lorlatinib significantly delayed disease progression compared to crizotinib.

The pooled analysis revealed that alectinib and brigatinib demonstrated superior TTD in fatigue symptom scores with a pooled HR of 0.71 (95% CI: 0.54 to 0.92) measured by EORTC QLQ-C30, compared to crizotinib. As a general and multifactorial symptom, fatigue may be influenced by disease burden and treatment-related tolerability. The observed improvements with alectinib and brigatinib might reflect better overall tolerability and sustained disease control, although further investigation is required to elucidate these associations [27]. However, the TTD was significantly shorter with alectinib than with crizotinib in dyspnea symptom measured by EORTC QLQ-LC13, with a HR of 1.76 (95% CI: 1.05 to 2.92). This finding could be associated with a higher incidence of grade ≥ 3 adverse event of pneumonia in alectinib compared to crizotinib (4.6% vs. 2.0%) as reported in the ALEX study [22]. This observation is also consistent with the adverse events listed in the alectinib label [28].

Our results indicated that next-generation ALK-TKIs, lorlatinib and brigatinib, showed significant improvement in physical and emotional functioning. Moreover, both drugs also demonstrated superior physical and emotional functioning scores compared to crizotinib, which may attribute to their anti-tumor activity against both extracranial and intracranial lesions [29], prolonged disease control, improved tolerability [4, 30], and reduced symptoms burden. Nonetheless, crizotinib was found to have a significant improvement in cognitive functioning over lorlatinib, aligning with the central nervous system (CNS) adverse events reported in the clinical trials of lorlatinib [31]. In the CROWN study, all-causality CNS adverse events occurred in 42% of patients in the lorlatinib group, of which 28% of events had cognitive effects [4]. Previous study suggested the neurotoxicity of lorlatinib could be partly attributed to its pharmacokinetics, specifically its ability to penetrate the blood–brain barrier and accumulate in the CNS and partial inhibition of trkB [32]. Brigatinib, on the other hand, reported significantly improved cognitive function as compared with crizotinib in ALTA-1L trial, with a between-arm change from baseline of 4.90 (95% CI: 1.70 to 8.10).

Moreover, lorlatinib and brigatinib also demonstrated significant improvement in symptoms of nausea and vomiting, fatigue, constipation, and appetite loss, compared with crizotinib. It is suspected that the improvement of HRQoL was correlated with adverse events reported in the trials. Regarding the reduction of patient-reported symptom burden, the pooled analysis using EORTC QLQ-C30 was consistent with the CROWN trial, showing lower rates of nausea (17% vs. 53%) and vomiting (13% vs. 39%) for lorlatinib compared to crizotinib, and similar consistency was observed for fatigue (30% vs. 33%), constipation (19% vs. 30%), and appetite loss (4% vs. 25%) [4]. In the ALTA-1L trial, the safety results of brigatinib also demonstrated consistency in symptoms with lower rates of nausea (26% vs. 56%), vomiting (18% vs. 39%), fatigue (18% vs. 20%), constipation (15% vs. 42%), and appetite loss (7% vs. 20%) compared to crizotinib [30].

Differences in HRQoL outcomes should be considered in the context of treatment distinctions. Compared with chemotherapy, ALK-TKIs provide targeted efficacy with generally fewer systemic toxicities [33]. Within ALK-TKIs, crizotinib shows more off-target effects and limited CNS activity, while next-generation agents (e.g., alectinib, brigatinib, lorlatinib) offer improved disease control but differ in toxicity profiles [21, 25, 34]. These distinctions might provide important context for interpreting the variation in HRQoL outcomes.

Evaluation of PROs in the published literature review and meta-analyses on ALK-TKIs was lacking until recently [12, 35, 36]. The literature review by Wang et al*.* suggested that brigatinib and lorlatinib significantly improved quality of life in comparison to crizotinib and chemotherapy [14]. Moreover, in a multifaced network meta-analysis (NMA) by Zhao et al*.*, PRO endpoints of ALK-TKIs among patients with advanced ALK-positive NSCLC were analyzed alongside efficacy and safety measurements [15]. Brigatinib appeared to be the best option on the dimension of PROs based on the results of EORTC QLQ-LC13 and EORTC QLQ-C30.

The findings of this systematic review and meta-analysis held significant implications for clinical practice in the field of *ALK*-positive NSCLC treatment. The observed overall improvement in HRQoL among patients treated with next-generation ALK-TKIs highlighted the potential advantages of these new agents, which may guide shared decision-making in treatment selection between clinicians and patients. HRQoL is a crucial aspect of cancer treatment, as it represents patients’ physical and emotional well-being, treatment adherence, and overall treatment outcomes [37, 38]. Recent NSCLC National Comprehensive Cancer Network and Chinese Society of Clinical Oncology guidelines [39, 40], as well as the China expert consensus on ALK-TKIs for advanced NSCLC [41], recommended the next-generation ALK-TKIs as the preferred first-line systemic therapy. The current study supported these guidelines with added evidence from the perspective of HRQoL, and it is hoped that the dimension of HRQoL evaluations may be incorporated into future guidelines.

In addition to the findings from the meta-analysis, we also identified several key factors that may help future studies to assess more comprehensive and comparable results on the effects of ALK-TKIs on HRQoL. First, there is a general lack of assessment of HRQoL in several ALK-TKIs’ trials, thus not all ALK-TKIs were included for each meta-analysis. This observation highlighted the underestimation of the importance of HRQoL in the field of ALK-TKIs trials. More attention and effort should be given to future research. Second, this study was limited to published data, which raised the possibility of publication bias. Trials with unfavorable HRQoL results were less likely to be reported, potentially leading to an overestimation of treatment benefits. Third, several different HRQoL instruments were used in the identified studies and limited themselves to be included in each meta-analysis. Although some instruments can be converted using established mapping algorithms [42–44], we were unable to perform this analysis due to lack of individual-level data. In addition to the use of different HRQoL instruments, we also observed diverse choices when assessing the domains (i.e., global, functioning, and symptoms), items (e.g., fatigue, constipation, and emotional functioning), and modalities (e.g., HR for TTD, between-arm change from baseline, change from baseline, and mean difference), and the precision measurements (e.g., 95% CI: standard deviation). Future studies may consider applying more consistent assessment measures. Lastly, due to insufficient number of studies, moderator analyses could not be conducted to explore sources of heterogeneity.

## Conclusions

These findings indicated that next-generation ALK-TKIs significantly delayed TTD in global health status compared to crizotinib. Brigatinib and alectinib had superior TTD in fatigue, while brigatinib and lorlatinib also outperformed crizotinib in global health status, physical and emotional functioning, and several symptom scores. The findings highlight the improved performance of next-generation ALK-TKIs in enhancing HRQoL for *ALK*-positive NSCLC patients. These benefits, aligning with recent studies and current clinical guideline recommendations, reinforced the position of next-generation ALK-TKIs as the preferred first-line treatment of *ALK*-positive NSCLC. Healthcare practitioners and other key stakeholders should therefore take into account the positive impact of next-generation ALK-TKIs on HRQoL when managing *ALK*-positive NSCLC patients in routine clinical settings. Future studies are needed to provide comprehensive and comparable assessments on HRQoL in clinical trials as HRQoL has increasingly playing a more important role in NSCLC patient management.

## Supplementary Information

Supplementary material

## Data Availability

The data underlying this manuscript will be shared on reasonable request to the corresponding author.

## References

[1] Sung, H., et al. (2021). Global cancer statistics 2020: GLOBOCAN estimates of incidence and mortality worldwide for 36 cancers in 185 countries. *CA: A Cancer Journal for Clinicians,**71*(3), 209–249. 33538338 10.3322/caac.21660

[2] Du, X., et al. (2018). ALK-rearrangement in non-small-cell lung cancer (NSCLC). *Thoracic Cancer,**9*(4), 423–430. 29488330 10.1111/1759-7714.12613 PMC5879058

[3] Peng, L., et al. (2022). Targeting ALK rearrangements in NSCLC: Current state of the art. *Frontiers in Oncology,**12*, Article 863461. 35463328 10.3389/fonc.2022.863461 PMC9020874

[4] Solomon, B. J., et al. (2024). Lorlatinib versus crizotinib in patients with advanced ALK-positive non-small cell lung cancer: 5-year outcomes from the phase III CROWN study. *Journal Of Clinical Oncology,**42*(29), 3400–3409. 38819031 10.1200/JCO.24.00581 PMC11458101

[5] Warsame, R., & D’Souza, A. (2019). Patient reported outcomes have arrived: A practical overview for clinicians in using patient reported outcomes in oncology. *Mayo Clinic Proceedings,**94*(11), 2291–2301. 31563425 10.1016/j.mayocp.2019.04.005 PMC6832764

[6] Kyte, D., et al. (2016). International Society for Quality of Life Research commentary on the draft European Medicines Agency reflection paper on the use of patient-reported outcome (PRO) measures in oncology studies. *Quality of Life Research,**25*(2), 359–362. 26275979 10.1007/s11136-015-1099-z

[7] Aaronson, N. K., et al. (1993). The European Organization for Research and Treatment of Cancer QLQ-C30: A quality-of-life instrument for use in international clinical trials in oncology. *Journal of the National Cancer Institute,**85*(5), 365–376. 8433390 10.1093/jnci/85.5.365

[8] Bergman, B., et al. (1994). The EORTC QLQ-LC13: a modular supplement to the EORTC Core Quality of Life Questionnaire (QLQ-C30) for use in lung cancer clinical trials. EORTC Study Group on Quality of Life. *European Journal of Cancer,**30A*(5), 635–642. 8080679 10.1016/0959-8049(94)90535-5

[9] Gnanasakthy, A., & DeMuro, C. R. (2024). The limitations of EQ-5D as a clinical outcome assessment tool. *Patient,**17*(3), 215–217. 38466537 10.1007/s40271-024-00683-w

[10] Hollen, P. J., et al. (1993). Quality of life assessment in individuals with lung cancer: Testing the Lung Cancer Symptom Scale (LCSS). *European Journal of Cancer,**29A*(Suppl 1), S51–S58. 10.1016/s0959-8049(05)80262-x 8381294

[11] Elliott, J., et al. (2020). ALK inhibitors for non-small cell lung cancer: A systematic review and network meta-analysis. *PLoS ONE,**15*(2), Article e0229179. 32074131 10.1371/journal.pone.0229179 PMC7029857

[12] Ma, H. C., et al. (2021). Comparative efficacy and safety of first-line treatments for advanced non-small cell lung cancer with ALK-rearranged: A meta-analysis of clinical trials. *BMC Cancer,**21*(1), 1278. 34836510 10.1186/s12885-021-08977-0 PMC8620528

[13] Tan, A. C., et al. (2023). First-line ALK inhibitors in treatment-naive advanced ALK rearranged non-small cell lung cancer: Systematic review and network meta-analysis. *Precision Cancer Medicine*. 10.21037/pcm-22-54

[14] Wang, W. Q., et al. (2023). A comprehensive clinical evaluation of first-line drugs for ALK-positive advanced non-small cell lung cancer. *Journal of Thoracic Disease,**15*(4), 1935–1947. 37197536 10.21037/jtd-23-380 PMC10183550

[15] Zhao, M., et al. (2024). Identifying optimal ALK inhibitors in first- and second-line treatment of patients with advanced ALK-positive non-small-cell lung cancer: A systematic review and network meta-analysis. *BMC Cancer,**24*(1), 186. 38331773 10.1186/s12885-024-11916-4 PMC10851546

[16] Tse, B. C., et al. (2020). Longitudinal health utilities, symptoms and toxicities in patients with ALK-rearranged lung cancer treated with tyrosine kinase inhibitors: A prospective real-world assessment. *Current Oncology,**27*(6), e552–e559. 33380870 10.3747/co.27.6563 PMC7755437

[17] Page, M. J., et al. (2021). The PRISMA 2020 statement: An updated guideline for reporting systematic reviews. *BMJ,**372*, Article n71. 33782057 10.1136/bmj.n71 PMC8005924

[18] Higgins, J. P., et al. (2003). Measuring inconsistency in meta-analyses. *BMJ (Clinical research ed.),**327*(7414), 557–560. 12958120 10.1136/bmj.327.7414.557 PMC192859

[19] Sidik, K., & Jonkman, J. N. (2005). Simple heterogeneity variance estimation for meta-analysis. *Journal of the Royal Statistical Society. Series C, Applied Statistics,**54*(2), 367–384.

[20] Shaw, A. T., et al. (2017). Ceritinib versus chemotherapy in patients with ALK-rearranged non-small-cell lung cancer previously given chemotherapy and crizotinib (ASCEND-5): A randomised, controlled, open-label, phase 3 trial. *The Lancet Oncology,**18*(7), 874–886. 28602779 10.1016/S1470-2045(17)30339-X

[21] Camidge, D. R., et al. (2021). Brigatinib versus crizotinib in ALK inhibitor-naive advanced ALK-positive NSCLC: Final results of phase 3 ALTA-1L trial. *Journal of Thoracic Oncology,**16*(12), 2091–2108. 34537440 10.1016/j.jtho.2021.07.035

[22] Mok, T., et al. (2020). Updated overall survival and final progression-free survival data for patients with treatment-naive advanced ALK-positive non-small-cell lung cancer in the ALEX study. *Annals Of Oncology,**31*(8), 1056–1064. 32418886 10.1016/j.annonc.2020.04.478

[23] Shaw, A. T., et al. (2020). First-line lorlatinib or crizotinib in advanced ALK-positive lung cancer. *The New England Journal of Medicine,**383*(21), 2018–2029. 33207094 10.1056/NEJMoa2027187

[24] Horn, L., et al. (2021). Ensartinib vs crizotinib for patients with anaplastic lymphoma kinase-positive non-small cell lung cancer: A randomized clinical trial. *JAMA Oncology,**7*(11), 1617–1625. 34473194 10.1001/jamaoncol.2021.3523 PMC8414368

[25] Solomon, B. J., et al. (2014). First-line crizotinib versus chemotherapy in ALK-positive lung cancer. *The New England Journal of Medicine,**371*(23), 2167–2177. 25470694 10.1056/NEJMoa1408440

[26] Marschner, N., et al. (2020). Association of disease progression with health-related quality of life among adults with breast, lung, pancreatic, and colorectal cancer. *JAMA Network Open,**3*(3), Article e200643. 32154886 10.1001/jamanetworkopen.2020.0643 PMC7064873

[27] Rocco, D., et al. (2019). Safety and tolerability of anaplastic lymphoma kinase inhibitors in non-small-cell lung cancer. *Drug Safety,**42*(2), 199–209. 30649741 10.1007/s40264-018-0771-y

[28] Genentech USA, I. *ALECENSA® (alectinib)*. (2021). July 20, 2023]; Available from: https://www.accessdata.fda.gov/drugsatfda_docs/label/2021/208434s010lbl.pdf

[29] Naito, T., Shiraishi, H., & Fujiwara, Y. (2021). Brigatinib and lorlatinib: Their effect on ALK inhibitors in NSCLC focusing on resistant mutations and central nervous system metastases. *Japanese Journal of Clinical Oncology,**51*(1), 37–44. 33147606 10.1093/jjco/hyaa192

[30] Camidge, D. R., et al. (2018). Brigatinib versus crizotinib in ALK-positive non-small-cell lung cancer. *The New England Journal of Medicine,**379*(21), 2027–2039. 30280657 10.1056/NEJMoa1810171

[31] Bauer, T. M., et al. (2019). Clinical management of adverse events associated with Lorlatinib. *The Oncologist,**24*(8), 1103–1110. 30890623 10.1634/theoncologist.2018-0380 PMC6693708

[32] Tanzilli, A., et al. (2024). EP.12B.03 cognitive impact of lorlatinib administration in advanced non small cell lung cancer with ALK and ROS1 fusions. *Journal of Thoracic Oncology,**19*(10), S641.

[33] Shaw, A. T., et al. (2013). Crizotinib versus chemotherapy in advanced ALK-positive lung cancer. *The New England Journal of Medicine,**368*(25), 2385–2394. 23724913 10.1056/NEJMoa1214886

[34] Peters, S., et al. (2017). Alectinib versus crizotinib in untreated ALK-positive non-small-cell lung cancer. *The New England Journal of Medicine,**377*(9), 829–838. 28586279 10.1056/NEJMoa1704795

[35] Breadner, D., et al. (2020). Efficacy and safety of ALK inhibitors in ALK-rearranged non-small cell lung cancer: A systematic review and meta-analysis. *Lung Cancer,**144*, 57–63. 32371261 10.1016/j.lungcan.2020.04.011

[36] Peng, L., et al. (2021). Efficacy and safety of first-line treatment strategies for anaplastic lymphoma kinase-positive non-small cell lung cancer: A Bayesian network meta-analysis. *Frontiers in Oncology,**11*, Article 754768. 34820326 10.3389/fonc.2021.754768 PMC8606689

[37] Montazeri, A., et al. (2001). Quality of life in lung cancer patients: As an important prognostic factor. *Lung Cancer,**31*(2–3), 233–240. 11165402 10.1016/s0169-5002(00)00179-3

[38] Trask, P. C., Hsu, M.-A., & McQuellon, R. (2009). Other paradigms: Health-related quality of life as a measure in cancer treatment: Its importance and relevance. *The Cancer Journal,**15*(5), 435–440. 19826365 10.1097/PPO.0b013e3181b9c5b9

[39] National Comprehensive Cancer Network. (2025). *NCCN Clinical Practice Guidelines in Oncology (NCCN Guidelines®): Non-Small Cell Lung Cancer [v.3.2025]*.

[40] Chinese Society of Clinical Oncology. (2024). *Guidelines of Chinese Society of Clinical Oncology (CSCO) Non-Small Cell Lung Cancer*.

[41] Chinese Association for Clinical, O., et al. (2024). *[China expert recommendations on anaplastic lymphoma kinase-tyrosine kinase inhibitors treatment for advanced non-small cell lung cancer (2024 edition)].* Zhonghua Yi Xue Za Zhi. *104*(7) 473–485. 10.3760/cma.j.cn112137-20231013-00729 38317359

[42] Arnold, D. T., et al. (2015). Testing mapping algorithms of the cancer-specific EORTC QLQ-C30 onto EQ-5D in malignant mesothelioma. *Health and Quality of Life Outcomes,**13*, 6. 25613110 10.1186/s12955-014-0196-y PMC4316600

[43] Jang, R. W., et al. (2010). Derivation of utility values from European Organization for Research and Treatment of Cancer Quality of Life-Core 30 questionnaire values in lung cancer. *Journal of Thoracic Oncology,**5*(12), 1953–1957. 21155140 10.1097/jto.0b013e3181f77a6a

[44] Khan, I., & Morris, S. (2014). A non-linear beta-binomial regression model for mapping EORTC QLQ- C30 to the EQ-5D-3L in lung cancer patients: A comparison with existing approaches. *Health and Quality of Life Outcomes,**12*, 163. 25388439 10.1186/s12955-014-0163-7 PMC4234877

[45] Blackhall, F., et al. (2014). Patient-reported outcomes and quality of life in PROFILE 1007: A randomized trial of crizotinib compared with chemotherapy in previously treated patients with ALK-positive advanced non-small-cell lung cancer. *Journal of Thoracic Oncology,**9*(11), 1625–1633. 25436797 10.1097/JTO.0000000000000318

[46] Blackhall, F., et al. (2017). Final results of the large-scale multinational trial PROFILE 1005: Efficacy and safety of crizotinib in previously treated patients with advanced/metastatic ALK-positive non-small-cell lung cancer. *ESMO Open,**2*(3), Article e000219. 29209525 10.1136/esmoopen-2017-000219 PMC5703388

[47] Wu, Y. L., et al. (2018). Results of PROFILE 1029, a phase III comparison of first-line crizotinib versus chemotherapy in East Asian patients with ALK-positive advanced non-small cell lung cancer. *Journal of Thoracic Oncology,**13*(10), 1539–1548. 29966800 10.1016/j.jtho.2018.06.012

[48] Soria, J. C., et al. (2017). First-line ceritinib versus platinum-based chemotherapy in advanced ALK-rearranged non-small-cell lung cancer (ASCEND-4): A randomised, open-label, phase 3 study. *The Lancet,**389*(10072), 917–929. 10.1016/S0140-6736(17)30123-X 28126333

[49] Nishio, M., et al. (2020). Final overall survival and other efficacy and safety results from ASCEND-3: Phase II study of ceritinib in ALKi-naive patients with ALK-rearranged NSCLC. *Journal of Thoracic Oncology,**15*(4), 609–617. 31778798 10.1016/j.jtho.2019.11.006

[50] Perol, M., et al. (2019). Patient-reported outcomes from the randomized phase III ALEX study of alectinib versus crizotinib in patients with ALK-positive non-small-cell lung cancer. *Lung Cancer,**138*, 79–87. 31654838 10.1016/j.lungcan.2019.10.002

[51] Garcia Campelo, M. R., et al. (2021). Health-related quality of life in the randomized phase III trial of brigatinib vs crizotinib in advanced ALK inhibitor-naive ALK + non-small cell lung cancer (ALTA-1L). *Lung Cancer,**155*, 68–77. 33744781 10.1016/j.lungcan.2021.03.005

[52] Mazieres, J., et al. (2022). Patient-reported outcomes from the randomized phase 3 CROWN study of first-line lorlatinib versus crizotinib in advanced ALK-positive non-small cell lung cancer. *Lung Cancer,**174*, 146–156. 36410210 10.1016/j.lungcan.2022.11.004

[53] Crino, L., et al. (2016). Multicenter phase II study of whole-body and intracranial activity with ceritinib in patients with ALK-rearranged non-small-cell lung cancer previously treated with chemotherapy and crizotinib: Results from ASCEND-2. *Journal of Clinical Oncology,**34*(24), 2866–2873. 27432917 10.1200/JCO.2015.65.5936

[54] Ou, S. I., et al. (2018). Patient-reported outcomes in a phase II, North American study of alectinib in patients with ALK-positive, crizotinib-resistant, non-small cell lung cancer. *ESMO Open,**3*(5), Article e000364. 30018815 10.1136/esmoopen-2018-000364 PMC6045737

[55] Kawata, A. K., et al. (2019). Converting EORTC QLQ-C30 scores to utility scores in the brigatinib ALTA study. *Journal of Medical Economics,**22*(9), 924–935. 31125274 10.1080/13696998.2019.1624080

[56] Lenderking, W. R., et al. (2019). Patient-reported outcomes and quality of life in advanced ALK+ non-small-cell lung cancer trial of brigatinib (ALTA). *Future Oncology,**15*(24), 2841–2855. 31364872 10.2217/fon-2019-0185

[57] Ou, S. I., et al. (2022). Efficacy of brigatinib in patients with advanced ALK-positive NSCLC who progressed on alectinib or ceritinib: ALK in Lung Cancer Trial of brigAtinib-2 (ALTA-2). *Journal of Thoracic Oncology,**17*(12), 1404–1414. 36096442 10.1016/j.jtho.2022.08.018

[58] Yang, Y., et al. (2020). Efficacy, safety, and biomarker analysis of ensartinib in crizotinib-resistant, ALK-positive non-small-cell lung cancer: A multicentre, phase 2 trial. *The Lancet Respiratory Medicine,**8*(1), 45–53. 31628085 10.1016/S2213-2600(19)30252-8

[59] Solomon, B. J., et al. (2018). Lorlatinib in patients with ALK-positive non-small-cell lung cancer: Results from a global phase 2 study. *The Lancet Oncology,**19*(12), 1654–1667. 30413378 10.1016/S1470-2045(18)30649-1

[60] Felip, E., et al. (2015). Impact of crizotinib on patient-reported general health status compared with chemotherapy in patients with no prior systemic treatment for advanced non-squamous ALK-positive non-small cell lung cancer (NSCLC). *Journal of Clinical Oncology*. 10.1200/jco.2015.33.15_suppl.8101

[61] Blackhall, F., Cappuzzo, F., Kim, D.W., Wu, Y., Solomon, B., Nakagawa, K., Mekhail, T., Paolini, J., Usari, T., Iyer, S., Reisman, A., Wilner, K., Tursi, J., Mok T. (2014). Impact of crizotinib on patient-reported symptoms and global quality of life (QoL) compared with platinum based chemotherapy in phase III study of treatment naïve advanced ALK-positive non-small cell lung cancer (NSCLC). *25*(supplement 4).

[62] Blackhall, F., et al. (2013). Impact of crizotinib on patient-reported general health status compared with single-agent chemotherapy in a phase III study of advanced ALK-positive non-small cell lung cancer (NSCLC). *European Journal of Cancer,**49*, S799–S800.

[63] Blackhall, F., et al. (2013). Impact of crizotinib on patient-reported symptoms and global quality of life (QoL) compared with chemotherapy in a phase III study of advanced alk-positive non-small cell lung cancer (NSCLC). *European Journal of Cancer,**49*, S795.

[64] Zhou, C., Kim, S. W., Reungwetwattana, T., Zhou, J., Zhang, Y., He, J., Yang, J. J., Cheng, Y., Lee, S. H., Bu, L., Xu, T., Yang, L., Wang, C., Morcos, P. N., Mitry, E., & Li, Z. (2018). Primary results of ALESIA: Phase III, randomised open-label study of alectinib (ALC) vs crizotinib (CRZ) in Asian patients (pts) with treatment-naïve ALK+ advanced non-small-cell lung cancer (NSCLC). *Annals of Oncology,**29*, ix174.

[65] Selvaggi, G., et al. (2021). FP14.12 Quality of life and subgroup analysis in a phase 3 randomized study of ensartinib vs crizotinib in ALK–positive NSCLC patients: EXalt3. *Journal of Thoracic Oncology,**16*(3), S232–S233.

[66] Liu, G., Reisman, A., Blackhall, F., & Mazieres, J. (2022). Health-related quality of life (HRQOL) in patients with ALK+ non-small cell lung cancer (NSCLC) in the phase III CROWN study. *Annals of Oncology,**33*(57), S1056.

[67] Mazieres, J., De Castro, J., Migliorino, M. R., Helland, Å., Dziadziuszko, R., Griesinger, F., Wolf, J., Zeaiter, A., Cardona, A., Balas, B., Karagiannis, T., Chlistalla, M., Smoljanovic, V., & Oh, I. (2017). Patient-reported outcomes and safety from the phase III ALUR study of Alectinib vs chemotherapy in pre-treated ALK+ NSCLC. *Journal of Thoracic Oncology* S1897.

[68] Greillier, L., et al. (2020). PCN17 health-related quality of life (HRQOL) of ALK-rearranged non-small cell lung cancer (NSCLC) patients treated in second line with alectinib. *Value in Health,**23*, S423.

