# Supplementary material for: Health-related quality of life among anaplastic lymphoma kinase (ALK)-positive non-small cell lung cancer (NSCLC) patients treated with first- and next-generation ALK tyrosine kinase inhibitors (TKIs): a systematic review and meta-analysis
Source: Qual Life Res. 2025 Nov 28;34(11):3073–92. doi: 10.1007/s11136-025-04088-6 (PMC12681492; doi:10.1007/s11136-025-04088-6)
Supplement: Supplementary file 1 — Supplementary material [file 11136_2025_4088_MOESM1_ESM.docx]

# **Appendix A. Supplementary data**

Supplementary Table 1. Search syntax in PubMed, Web of Science, Embase, and ClinicalTrials.gov^b^

| **Database** | **#** | **Topics** | **Search Strings** | **Number of records^a^** |
| --- | --- | --- | --- | --- |
| **PubMed** | **1** | **HRQoL** | "HRQoL"[All Fields] OR "quality of life"[MeSH Terms] OR "quality of life"[All Fields] OR "QoL"[All Fields] OR "EQ-5D"[All Fields] OR "QLQ-C30"[All Fields] OR "QLQ-LC13"[All Fields] | 500,625 |
|  | **2** | **NSCLC** | "carcinoma, non small cell lung"[MeSH Terms] OR "non small cell lung cancer"[Title/Abstract] OR "NSCLC"[Title/Abstract] | 110,313 |
|  | **3** | ***ALK* positive** | "alk rearrange*"[Title/Abstract] OR "alk alteration*"[Title/Abstract] OR "alk mutat*"[Title/Abstract] OR "alk resist*"[Title/Abstract] OR "alk positive*"[Title/Abstract] OR "Anaplastic lymphoma kinase"[MeSH Terms] OR "anaplastic lymphoma kinase"[Title/Abstract] | 9,042 |
|  | **4** | **ALK-TKIs** | "alk inhibitor*"[Title/Abstract] OR "crizotinib"[All Fields] OR "ceritinib"[All Fields] OR "alectinib"[All Fields] OR "brigatinib"[All Fields] OR "lorlatinib"[All Fields] OR "ensartinib"[All Fields] OR "iruplinalkib"[All Fields] OR "envonalkib"[All Fields] OR "ALK-TKIs"[Title/Abstract] OR "alk tyrosine kinase inhibitors"[Title/Abstract] | 5,660 |
|  | **/** | **Full strings** | #1 AND #2 AND #3 AND #4 | **101** |
| **Web of Science** | **1** | **HRQoL** | ALL = ("HRQoL" OR "quality of life" OR "QoL" OR "EQ-5D" OR "QLQ-C30" OR "QLQ-LC13") | 654,378 |
|  | **2** | **NSCLC** | TS = ("non small cell lung cancer" OR "NSCLC") | 136,492 |
|  | **3** | ***ALK* positive** | TS = ("alk rearrange*" OR "alk alteration*" OR "alk mutat*" OR "alk resist*" OR "alk positive*" OR "anaplastic lymphoma kinase") | 9,789 |
|  | **4** | **ALK-TKIs** | TS = ("alk inhibitor*" OR "crizotinib" OR "ceritinib" OR "alectinib" OR "brigatinib" OR "lorlatinib" OR "ensartinib" OR "iruplinalkib" OR "envonalkib" OR "ALK-TKIs" OR "alk tyrosine kinase inhibitors") | 8,789 |
|  | **/** | **Full strings** | #1 AND #2 AND #3 AND #4 | **135** |
| **Embase** | **1** | **HRQoL** | 'hrqol' OR 'health related quality of life' OR 'quality of life'/exp OR 'quality of life' OR 'eq-5d' OR 'qlq-c30' OR 'qlq-lc13' | 654,378 |
|  | **2** | **NSCLC** | 'non small cell lung cancer':ab,ti,kw OR 'nsclc':ab,ti,kw OR 'non small cell lung cancer'/exp | 136,492 |
|  | **3** | ***ALK* positive** | 'alk rearrange*':ab,ti,kw OR 'alk alteration*':ab,ti,kw OR 'alk mutat*':ab,ti,kw OR 'alk resist*':ab,ti,kw OR 'alk positive*':ab,ti,kw OR 'anaplastic lymphoma kinase'/exp OR 'anaplastic lymphoma kinase':ab,ti,kw | 9,789 |
|  | **4** | **ALK-TKIs** | 'alk inhibitor*':ab,ti,kw OR 'crizotinib' OR 'ceritinib' OR 'alectinib' OR 'brigatinib' OR 'lorlatinib' OR 'ensartinib' OR "iruplinalkib" OR "envonalkib" OR 'alk-tkis':ab,ti,kw OR 'alk tyrosine kinase inhibitors':ab,ti,kw | 8,789 |
|  | **/** | **Full strings** | #1 AND #2 AND #3 AND #4 | **135** |
| **ClinicalTrials.gov** | **/** | **Outcome Measures: HRQoL** | HRQoL OR quality of life OR QoL OR EQ-5D OR QLQ-C30 OR QLQ-LC13 | **43** |
|  | **/** | **Condition or disease: NSCLC** | Non Small Cell Lung Cancer OR NSCLC |  |
|  | **/** | **Other terms: *ALK* positive** | alk rearrange OR alk alteration OR alk mutation OR alk resist OR alk positive OR anaplastic lymphoma kinase |  |
|  | **/** | **Intervention/treatment: ALK-TKIs** | Crizotinib OR Brigatinib OR Alectinib OR Ceritinib OR Ensartinib OR Lorlatinib OR Iruplinalkib OR Envonalkib OR ALK-TKI OR ALK inhibitor |  |

Abbreviations: ALK: anaplastic lymphoma kinase; ALK-TKI: anaplastic lymphoma kinase-tyrosine kinase inhibitor; QLQ-LC13: Quality of Life Questionnaire Lung Cancer 13; QLQ-C30: Quality of Life Questionnaire Core 30; EQ-5D: European Quality of Life Five Dimension; HRQoL: health-related quality of life

^a^ The search was conducted on 14^th^ January, 2025 and will be updated close to manuscript submission.

^b^ Study type is limited to interventional studies (clinical trials)

Supplementary Table 2. Details of methodology of HRQoL instruments and HRQoL results presentation

|  | Full-text articles (N=20)  n (%) | Abstracts/posters (N=9)  n (%) |
| --- | --- | --- |
| HRQoL instrument (measured) (not mutually exclusive) |  |  |
| EORTC-QLQ-C30 | 20 (100%) | 6 (66.7%) |
| EORTC-QLQ-LC13 | 18 (90.0%) | 5 (55.6%) |
| EQ-5D | 7 (35.0%) | 4 (44.4%) |
| LCSS | 5 (25.0%) | / |
| HRQoL instrument (reported) (not mutually exclusive) |  |  |
| EORTC-QLQ-C30 | 18 (90.0%) | 5 (55.6%) |
| EORTC-QLQ-LC13 | 16 (80.0%) | 3 (33.3%) |
| EQ-5D | 5 (25.0%) | 3 (33.3%) |
| LCSS | 5 (25.0%) | / |
| Modality of HRQoL results presentation (not mutually exclusive) |  |  |
| Time to deterioration |  |  |
| Time to deterioration (global) | 4 (20.0%) | 3 (33.3%) |
| Time to deterioration (functioning) | 3 (15.0%) | 1 (11.1%) |
| Time to deterioration (symptoms) | 11 (55.0%) | 3 (33.3%) |
| Time to improvement |  |  |
| Time to improvement (global) | / | / |
| Time to improvement (functioning) | / | / |
| Time to improvement (symptoms) | 1 (5.0%) | / |
| Duration of improvement |  |  |
| Duration of improvement (global) | 1 (5.0%) | / |
| Duration of improvement (functioning) | / | / |
| Duration of improvement (symptoms) | / | / |
| Descriptive proportion of improvement/stability/deterioration |  |  |
| Descriptive proportion of improvement/stability/deterioration (global) | 6 (30.0%) | 3 (33.3%) |
| Descriptive proportion of improvement/stability/deterioration (functioning) | 4 (20.0%) | 3 (33.3%) |
| Descriptive proportion of improvement/stability/deterioration (symptoms) | 5 (25.0%) | 2 (22.2%) |
| Change from baseline |  |  |
| Change from baseline (global) | 11 (55.0%) | 2 (22.2%) |
| Change from baseline (functioning) | 6 (30.0%) | 1 (11.1%) |
| Change from baseline (symptoms) | 9 (45.05%) | 1 (11.1%) |
| Between-arm change from baseline |  |  |
| Between-arm change from baseline (global) | 5 (25.0%) | / |
| Between-arm change from baseline (functioning) | 5 (25.0%) | / |
| Between-arm change from baseline (symptoms) | 5 (25.0%) | / |
| Mean HRQoL score |  |  |
| Mean HRQoL score (global) | 3 (15.0%) | 4 (44.4%) |
| Mean HRQoL score (functioning) | / | / |
| Mean HRQoL score (symptoms) | 1 (5.0%) | / |
| Mean treatment difference |  |  |
| Mean treatment difference (global) | 2 (10.0%) | 1 (11.1%) |
| Mean treatment difference (functioning) | / | / |
| Mean treatment difference (symptoms) | 1 (5.0%) | / |
| Relative risk ratios for improvement rates |  |  |
| Relative risk ratios for improvement rates (global) | / | / |
| Relative risk ratios for improvement rates (functioning) | / | / |
| Relative risk ratios for improvement rates (symptoms) | / | 1 (11.1%) |
| Number of modalities of HRQoL results presentation^a^ |  |  |
| 1 | / | 2 (22.2%) |
| 2 | 4 (20.0%) | 2 (22.2%) |
| 3 | 5 (25.0%) | 2 (22.2%) |
| 4 | 6 (30.0%) | 2 (22.2%) |
| ≥ 5 | 5 (25.0%) | 1 (11.1%) |

Abbreviations: EORTC QLQ-LC13: European Organization for Research and Treatment of Cancer Quality of Life Questionnaire Lung Cancer 13; EORTC QLQ-C30: European Organization for Research and Treatment of Cancer Quality of Life Questionnaire Core 30; EQ-5D: European Quality of Life Five Dimension; HRQoL: health-related quality of life; LCSS: Lung Cancer Symptom Scale

^a^ Modalities: time to deterioration; time to improvement; duration of improvement; descriptive proportion of improvement/stability/deterioration; change from baseline; between-arm change from baseline; mean HRQoL score; mean treatment difference; relative risk ratios for improvement rates

Supplementary Table 3. Cochrane Risk of Bias of the 20 full-text publications
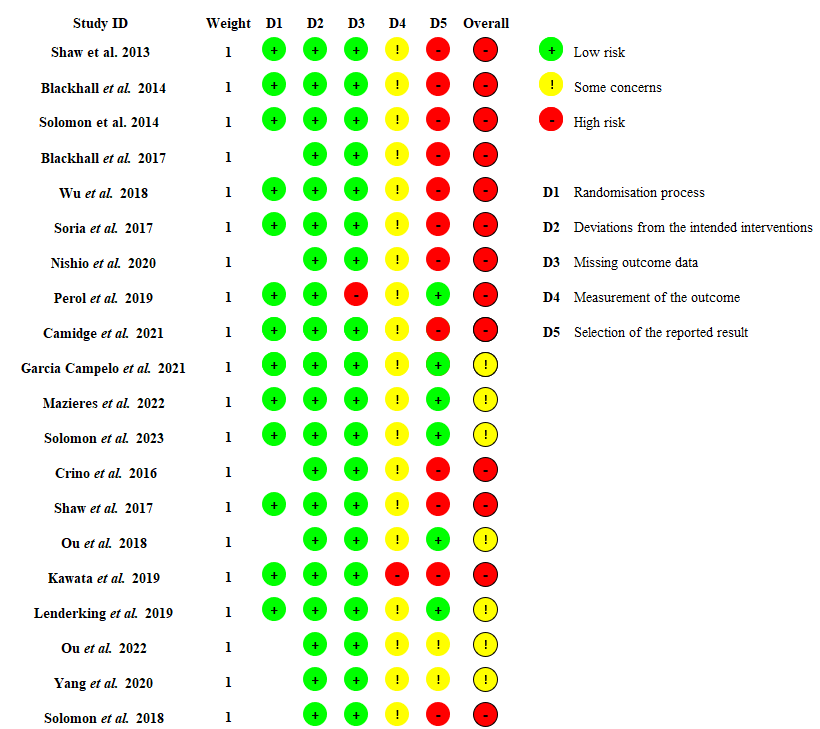


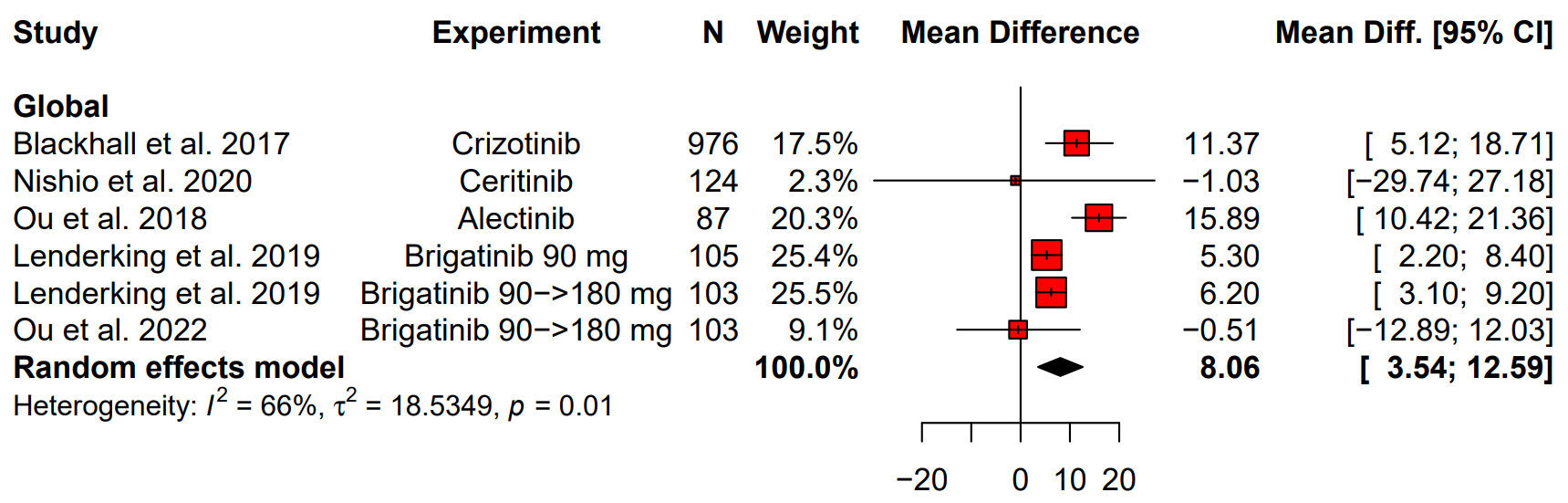


Supplementary Figure 1. Change from baseline of global health status comparing among ALK-TKIs using EORTC QLQ-C30

Abbreviations: ALK-TKIs: anaplastic lymphoma kinase-tyrosine kinase inhibitors; Cl: confidence level; EORTC QLQ-C30: European Organization for Research and Treatment of Cancer Quality of Life Questionnaire Core 30; HR: hazard ratio; N: number; mg: milligram


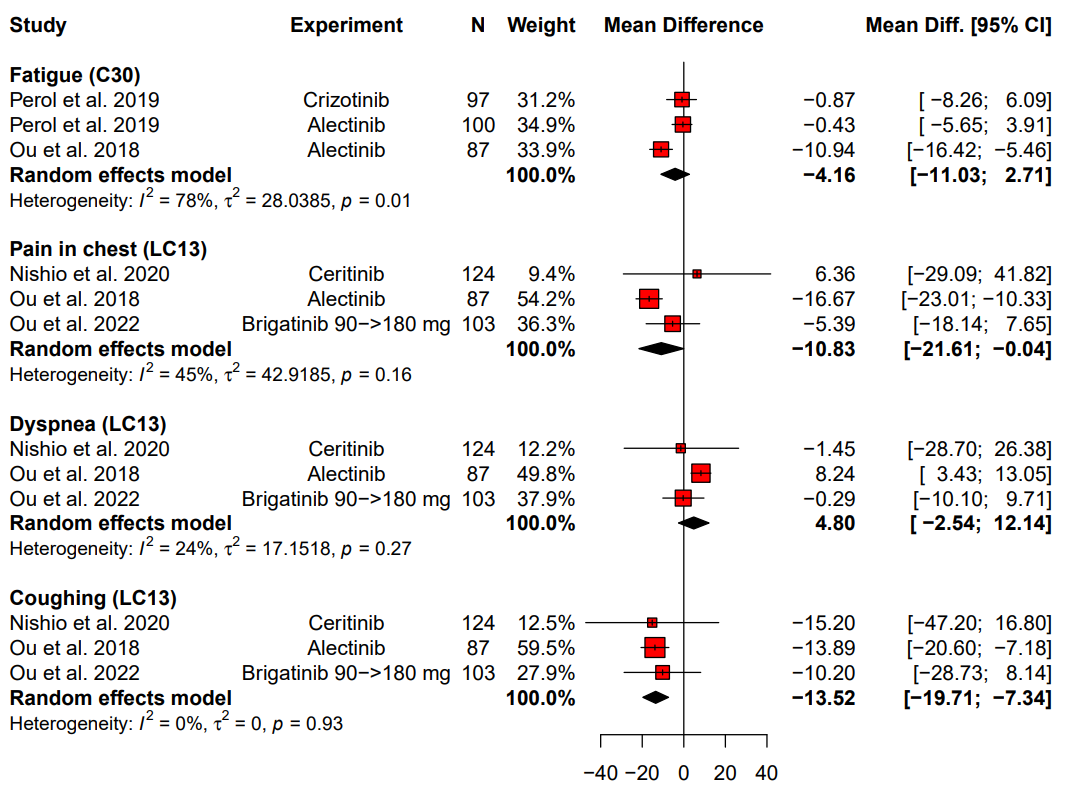


Supplementary Figure 2. Change from baseline of symptom score comparing among next generation ALK-TKIs using EORTC QLQ-C30 and EORTC QLQ-LC13

Abbreviations: ALK-TKIs: anaplastic lymphoma kinase-tyrosine kinase inhibitors; Cl: confidence level; EORTC QLQ-C30: European Organization for Research and Treatment of Cancer Quality of Life Questionnaire Core 30; EORTC QLQ-LC13: EORTC QLQ-Lung Cancer 13; HR: hazard ratio; N: number; mg: milligram


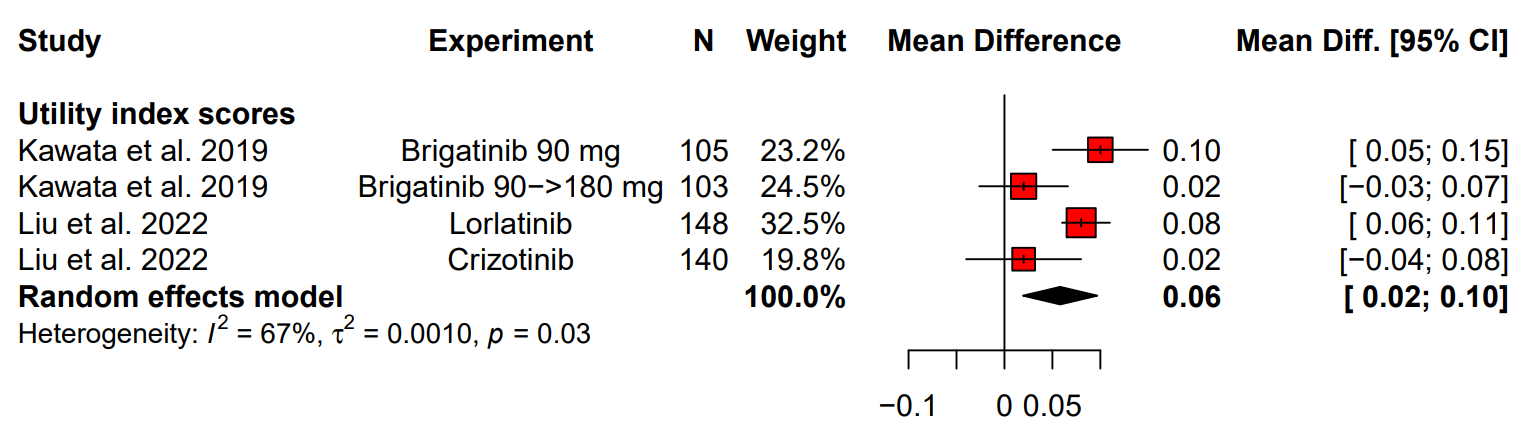


Supplementary Figure 3. Change from baseline of utility index scores comparing among next generation ALK-TKIs using EQ-5D

Abbreviations: ALK-TKIs: anaplastic lymphoma kinase-tyrosine kinase inhibitors; Cl: confidence level; EQ-5D: European Quality of Life Five Dimension; HR: hazard ratio; N: number; mg: milligram


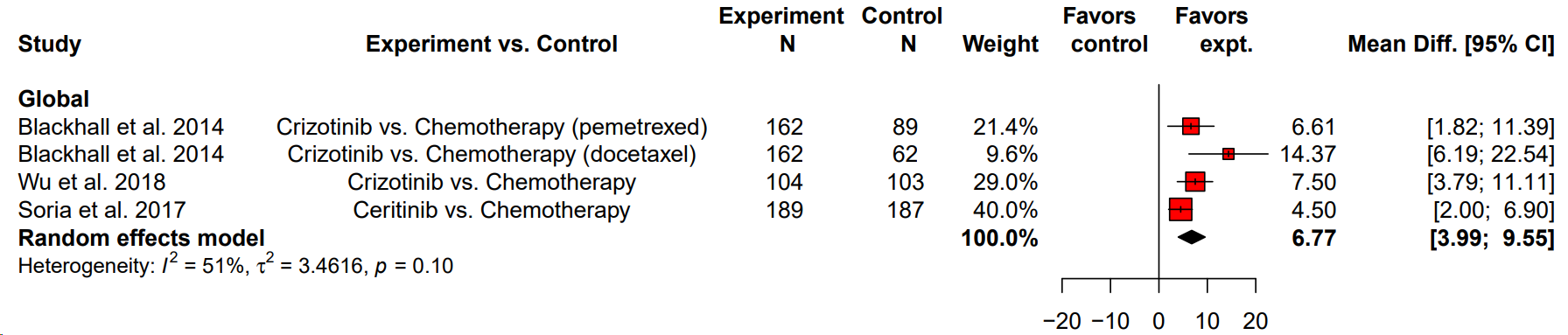


Supplementary Figure 4. Between-arm change from baseline of global health status score comparing crizotinib and ceritinib to chemotherapy using EORTC QLQ-C30

Abbreviations: Cl: confidence level; EORTC QLQ-C30: European Organization for Research and Treatment of Cancer Quality of Life Questionnaire Core 30; N: number


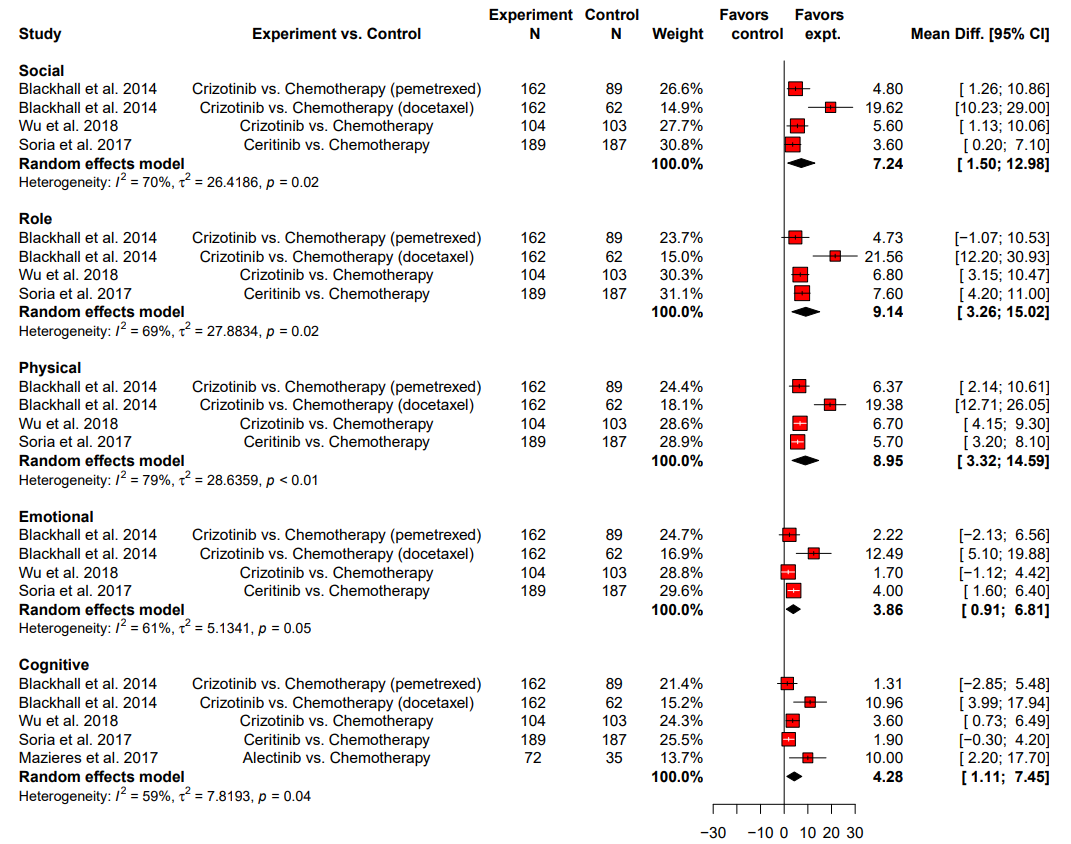


Supplementary Figure 5. Between-arm change from baseline of functioning score comparing crizotinib and ceritinib to chemotherapy using EORTC QLQ-C30

Abbreviations: Cl: confidence level; EORTC QLQ-C30: European Organization for Research and Treatment of Cancer Quality of Life Questionnaire Core 30; expt: experiment; N: number.


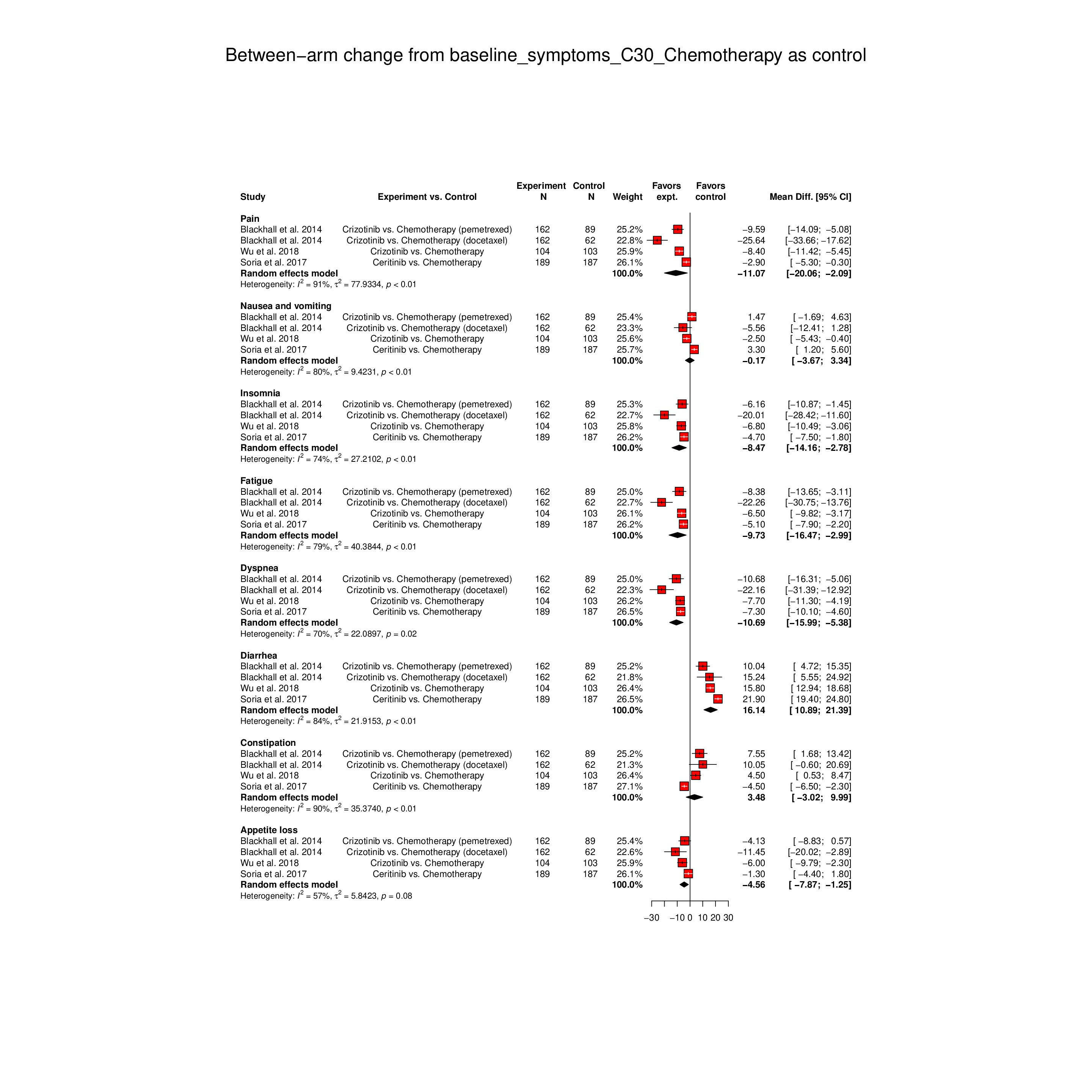


Supplementary Figure 6. Between-arm change from baseline of symptom score comparing crizotinib and ceritinib to chemotherapy using EORTC QLQ-C30

Abbreviations: Cl: confidence level; EORTC QLQ-C30: European Organization for Research and Treatment of Cancer Quality of Life Questionnaire Core 30; expt: experiment; N: number.


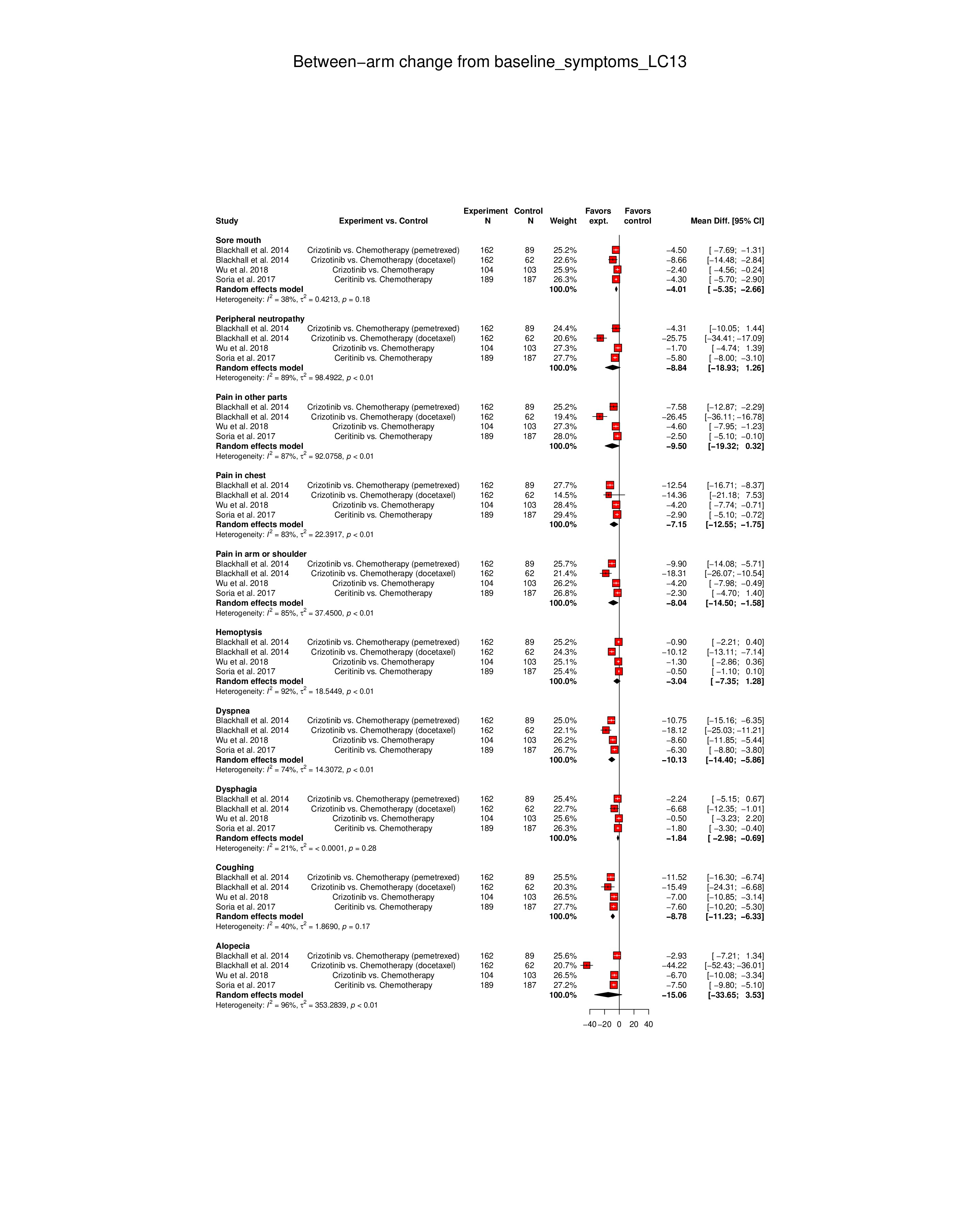


Supplementary Figure 7. Between-arm change from baseline of symptom score comparing crizotinib and ceritinib to chemotherapy using EORTC QLQ-LC13

Abbreviations: Cl: confidence level; EORTC QLQ-LC13: European Organization for Research and Treatment of Cancer Quality of Life Questionnaire Lung Cancer 13; HR: hazard ratio; N: number.


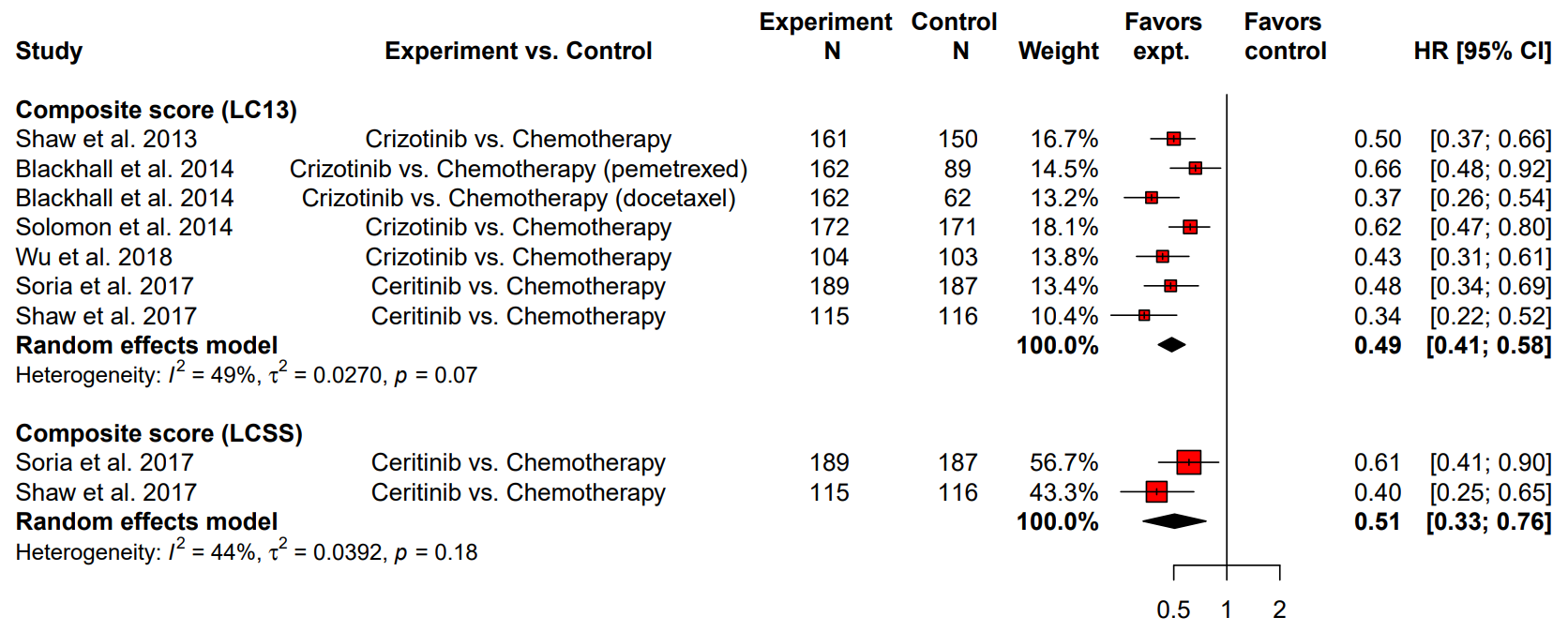


Supplementary Figure 8. Hazard ratios for time to deterioration of symptom composite score comparing crizotinib and ceritinib to chemotherapy using EORTC QLQ-LC13 and LCSS

Abbreviations: Cl: confidence level; EORTC QLQ-LC13: European Organization for Research and Treatment of Cancer Quality of Life Questionnaire Lung Cancer 13; expt: experiment; LCSS: Lung Cancer Symptom Scale; HR: hazard ratio; N: number.


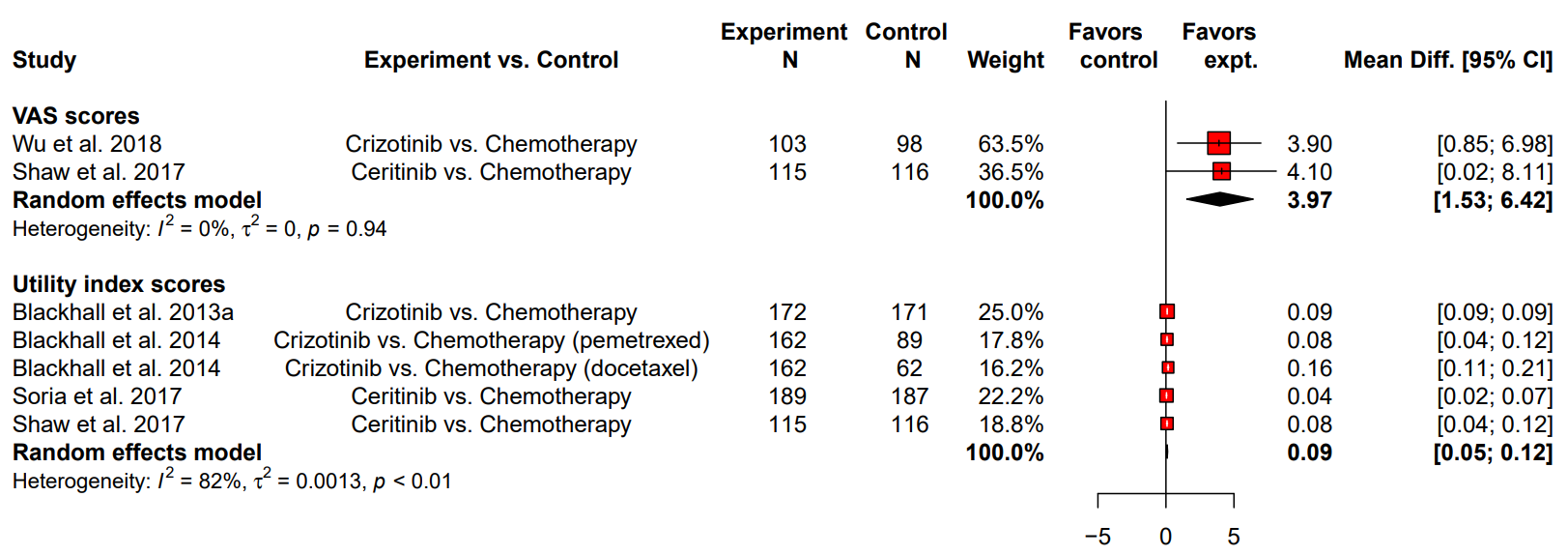


Supplementary Figure 9. Differences of mean VAS scores and utility index scores at the end of observation comparing crizotinib and ceritinib to chemotherapy using EQ-5D

Abbreviations: Cl: confidence level; expt: experiment; N: number; EQ-5D: European Quality of Life Five Dimension; VAS: visual analog scale.
